# HIV epidemic in fishing communities in Uganda: A scoping review

**DOI:** 10.1371/journal.pone.0249465

**Published:** 2021-04-01

**Authors:** Patou Masika Musumari, Teeranee Techasrivichien, Kriengkrai Srithanaviboonchai, Rhoda K. Wanyenze, Joseph K. B. Matovu, Hemant Poudyal, S. Pilar Suguimoto, Saman Zamani, Arunrat Tangmunkongvorakul, Masako Ono-Kihara, Masahiro Kihara

**Affiliations:** 1 Global Health Interdisciplinary Unit, Center for the Promotion of Interdisciplinary Education and Research, Kyoto University, Kyoto City, Kyoto, Japan; 2 International Institute of Socio-epidemiology, Kyoto City, Kyoto, Japan; 3 Research Institute for Health Sciences, Chiang Mai University, Chiang Mai City, Chiang Mai, Thailand; 4 Department of Community Medicine, Faculty of Medicine, Chiang Mai University, Chiang Mai City, Thailand; 5 Makerere University School of Public Health, Kampala, Uganda; 6 Busitema University Faculty of Health Sciences, Mbale, Uganda; 7 Centre for Medical Education and Department of Diabetes, Endocrinology and Nutrition, Graduate School of Medicine, Kyoto University, Kyoto City, Japan; 8 The Global Funds to Fight AIDS, Tuberculosis and Malaria, Geneva, Switzerland; 9 Department of Health Informatics, Kyoto University School of Public Health, Kyoto City, Japan; International AIDS Vaccine Initiative, UNITED STATES

## Abstract

**Background:**

Fishing communities in many Sub-Saharan African countries are a high-risk population group disproportionately affected by the HIV epidemic. In Uganda, literature on HIV in fishing communities has grown extensively since the first country’s documented case of HIV in a fishing community in 1985. The current study describes the status of the HIV burden, prevention, and treatment in Ugandan fishing communities.

**Method:**

This scoping review was conducted based on the York Framework outlined by Arksey and O’Malley. We searched the PubMed, Embase, and Web of Science databases to identify relevant quantitative and qualitative studies on HIV incidence, HIV prevalence, HIV-related risk factors, HIV testing, antiretroviral therapy coverage and adherence, and interventions to improve treatment outcomes and reduce HIV risk factors.

**Results & conclusion:**

We identified 52 papers and 2 reports. Thirty-four were quantitative, 17 qualitative, and 3 had a mixed-methods design. Eleven studies reported on the prevalence of HIV and 8 on HIV incidence; 9 studies documented factors associated with HIV incidence or HIV positive status; 10 studies reported on HIV testing coverage and/or associated factors; 7 reported on antiretroviral therapy coverage/adherence/outcomes; and 1 study reported on the impact of combination HIV interventions in fishing communities. This scoping review revealed a significant lack of evidence in terms of what works in HIV prevention and for improving adherence to ART, in contrast to the relatively large amount of evidence from observational quantitative and qualitative studies on HIV prevalence, incidence and related risk factors in Ugandan fishing communities. Intervention studies are urgently needed to fill the current evidence gaps in HIV prevention and ART adherence.

## Introduction

Fisheries are among the most important natural resources in Africa and vital for the economy and survival of many communities in several African countries. Estimates from 2011 indicate that the fisheries sector, including inland fishing, artisanal marine fishing, and industrial marine fishing contributed US$21.254 billion to the local economies, constituting 1.11% of the gross domestic product (GDP) of all African countries [1]. Approximately 12,269,000 people are employed in this sector in Africa, of which 6,147,000 (50.1%) are fishers and 5,202,000 (42.3%) are fish processors [1]. Almost 9 million (73.3%) people are employed in the inland fisheries and artisanal marine fisheries [1]. In the major inland fishing countries such as Uganda, Tanzania, and Kenya, the fisheries sector contributes up to 2.5% [2], 2.4% [3], and 0.54% [4] of the GDP, and provides employment to at least 1.2 million, 4 million, and 2 million people, respectively [2–4].

In Uganda, fisheries represent a rapidly growing sector that contributes to national food security, employment, and earnings from exports. Fish and fish products are Uganda’s second largest export. Investment in the fisheries sector was estimated at US$200 million in 2008 [5]. According to Uganda’s Ministry of Agriculture, Animal Industry and Fisheries [6] and the World Bank [7], roughly 18% of Uganda’s total surface area is covered in water (44,000 km of 241,000 km). Major lakes include Victoria (68,800 km^2^), Albert (5,335 km^2^), Kyoga (2,047 km^2^), Edward (2,300 km^2^) and George (250 km^2^). The first 3 lakes combined contribute about 95% of the country’s total annual fish catch, while Lake Victoria alone contributes about half of the total annual catch. Other fishing locations come from 160 minor lakes, rivers, floodplains, swamps and manmade fishing ponds.

Residents of fishing communities are one of the most-at-risk groups for HIV in Sub-Saharan Africa [8, 9]. Frequent mobility, transactional and commercial sex, multiple sexual partners, high consumption of alcohol, poor health infrastructure, and limited access to health services are reported among the main factors shaping the HIV epidemic in finishing communities [10–16]. In Uganda, fishing communities have been identified as key populations in the epidemic, along with other groups such as commercial sex workers, uniformed services, truck drivers, and men who have sex with men [17]. Fishing communities are composed of several groups: fisherfolk (fishermen, fish processors, fishmongers, boat makers and repairers, etc.), shopkeepers, bar/restaurant/lodge owners and workers, and commercial sex workers (CSW) who follow the fishermen as they move in search of better fish yields [12, 18, 19].

The first AIDS cases in Uganda were documented in fishing villages on the shores of Lake Victoria in 1985 [20]. Since then, there has been significant interest in understanding the dynamics of HIV transmission in Ugandan fishing communities, as reflected by an increasing number of published studies in the past two decades. The current study is a scoping review that aims to map the HIV situation in Ugandan fishing communities by pulling together studies published over the two decades in Ugandan fishing communities. The review intends to draw a comprehensive picture of the HIV risk factors, HIV prevalence and incidence, access to antiretroviral treatment (ART), adherence to ART and retention in care, and ART outcomes in fishing communities in Uganda. This review also intends to highlight the importance of locally-defined key populations, such as fisherfolk in Uganda, beyond the generic definition of key populations which include men who have sex with men, sex workers, transgender people, people who inject drugs, and prisoners and incarcerated people [21]. The focus of this scoping review on Uganda is to identify country-specific gaps in research regarding HIV prevention and treatment and care in Ugandan fishing communities. Such a country-level synthesis could be a useful resource for policy makers, researchers and other key stakeholders for improving the research and policy landscape of HIV infection in fishing communities.

## Methodologies

A scoping review, unlike a systematic review, broadly surveys the literature, but does not evaluate articles for methodological quality [22]. This scoping review was conducted based on the York Framework outlined by Arksey and O’Malley [23], which includes the following steps: identification of the research question to be addressed; identification of studies relevant to the research question; selection of studies to include in the review; charting of information and data within the included studies; and collating, summarizing and reporting results of the review.

### Identification of the research question

**Fig 1:**
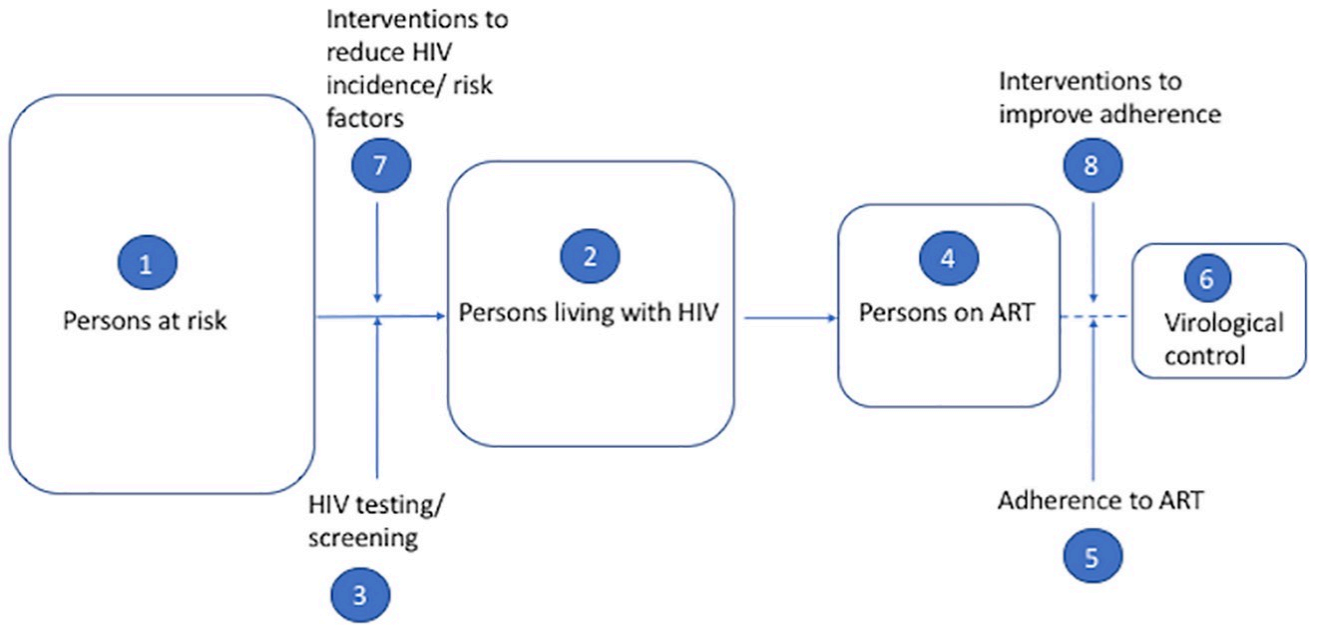
Conceptual framework. ART: antiretroviral therapy.

The review addressed the following research questions (RQ) depicted in our conceptual framework (Fig 1): 1) What are the risk factors for HIV infection in fishing communities in Uganda?; 2) What are the prevalence and incidence of HIV infection in Ugandan fishing communities?; 3) What is the prevalence of and factors that influence HIV testing in Ugandan fishing communities?; 4) What is the level of antiretroviral treatment (ART) coverage in fishing communities in Uganda?; 5) What is the level of adherence to ART and factors associated with non-adherence to ART among HIV-infected fisherfolk on ART?; 6) What is the proportion of HIV positive fisherfolk on ART who have achieved virological control?; 7) What is the current evidence on interventions to reduce HIV risk factors in fishing communities?; and 8) What is the current evidence on interventions to improve HIV treatment outcomes in fishing communities?

### Identification and selection of relevant studies

Searches were performed in December 2020 using the PubMed, Embase, and Web of Science databases. We also searched the reference lists of all the selected studies, as well as the grey literature (using the Google’s Internet search engine) to identify reports and other relevant documents. The inclusion criteria consisted of full papers or reports written in English, explicitly targeting fishing communities in Uganda and providing information relevant for the above research questions. There was no restriction on publication date. We searched for all available articles until December 2020. In terms of the study design, we included both quantitative (cross-sectional, case-control, prospective, and interventional design) and qualitative studies. The exclusion criteria were: no full-text available; reviews (systematic reviews; scoping reviews; literature reviews); and study protocols. The search strategy for PubMed is presented in Table 1. The search strategies for Embase and Web of Science are available in S1 Appendix.

**Table 1:** PubMed search strategy.

Conceptual areas	MeSH terms and free text terms	Boolean connectors
Fishing communities	“Fisherm*” [keyword] OR “fisherfolk” [keyword] OR “seafarer” [keyword] OR “seamen” [keyword] OR “boatmen” [keyword] OR “fishing communit*” [keyword] OR OR “fisherfolk communit*” [keyword] OR “fisheries” [keyword] OR “fish farm” [keyword]	AND
HIV/AIDS	“HIV” [MeSH] OR “HIV infections” [MeSH] OR “acquired immunodeficiency syndrome” [MeSH] OR “hiv infections/epidemiology” [keyword] OR “hiv epidemiology” [keyword]	AND
HIV risk factors	“Risk factors” [MeSH] OR sex factors [MeSH] OR “sexual behavior” [MeSH] OR “unsafe sex” [MeSH] OR “condoms” [MeSH] OR “sexual partners” [MeSH] OR “risk-taking” [MeSH] OR “predictor*” [keyword] OR “determinant*” [keyword] OR “health risk behaviors” [keyword] OR “risk-taking” [keyword] OR “risky sexual behavior” [keyword] OR “risky behavior” [keyword] OR “associated factor*” [keyword] OR “sexually transmitted diseases” [MeSH] OR “circumcision, male” [MeSH] OR “male circumcision*” [MeSH] OR “health knowledge, attitudes, practice”	OR
Prevalence/Incidence	“Prevalence” [MeSH] OR “hiv prevalence” [keyword] OR “incidence” [MeSH]	OR
HIV testing	“hiv infections/diagnosis” [MeSH] OR “mass screening” OR “hiv testing” [MeSH] OR “voluntary counsel*” [keyword] and “testing” [keyword] OR “hiv testing and counsel* [keyword] OR “hiv infections/diagnosis” [keyword] OR “serologic test” [MeSH] OR “point-of-care testing* [MeSH]	OR
Antiretroviral treatment	OR “hiv prevalence” [keyword] OR “Antiretroviral Therapy, Highly Active” [MeSH] OR “anti-HIV agents” [MeSH] OR “antiretroviral therapy” [keyword] OR “hiv infections/drug therapy [MeSH] OR “antiretroviral therapy” [keyword] OR “HIV control” [keyword]	OR
Adherence/compliance	“Medication adherence” [MeSH] OR “patient compliance” [MeSH]	AND
Study design	“cross-sectional studies” [MeSH] OR “health surveys” [MeSH] OR “cohort studies” [MeSH] OR “case-control studies” [MeSH] OR “clinical study” [MeSH] OR “qualitative research” [MeSH] OR “hiv intervention”[keyword] OR “HIV prevention”[keyword]	
Country	Uganda	

### Data extraction

Publication details from all three databases were exported into an Excel file and merged to select eligible citations. Duplicates were removed manually by PMM. Two investigators (PMM and TT) independently screened the publication titles and selected titles that addressed the target population (fishing communities) and/or the aforementioned research questions then examined the abstracts and full-texts articles of the selected titles and determined selection based on the inclusion and exclusion criteria. Data were extracted using a standardized form, which included study authors, publication year, study period, study design, study location, sample size, and the relevant research question the paper or report addressed.

### Collating, summarizing, and reporting results

The results were organized based on thematic areas as guided by the research questions. The thematic categories included the prevalence and incidence of HIV; the risk factors of HIV infection; the prevalence of HIV testing; the level of coverage and adherence to ART; and interventions studies to reduce HIV risks or improve HIV treatment outcomes. The prevalence and incidence rates of HIV were presented as reported in the selected studies, and stratification by age and sex was reported as per data availability. Risk factors were identified in accordance to those previously reported in the HIV literature and those that emerged from the selected studies: sexual risk behaviors (multiple sexual partners; unprotected sex; commercial/transactional sex; other sexually transmitted infections); knowledge of and attitudes toward HIV; substance use (e.g. alcohol); and socio-economic and structural factors (education level; age; occupational mobility). We presented both the prevalence of and the measures of association and confidence intervals of risk factors significantly associated with HIV infection. We analyzed intervention studies in light of their effects on HIV incidence, HIV risk factors, and HIV treatment outcomes (viral suppression; adherence to ART).

## Results

This section presents findings on the characteristics of the selected studies; characteristics of fishing communities; prevalence and incidence of HIV; HIV risk factors; prevalence of and factors that influence HIV testing; antiretroviral treatment coverage and adherence to ART, and interventions to improve HIV treatment outcomes in fishing communities.

### Characteristics of the selected studies

**Fig 2:**
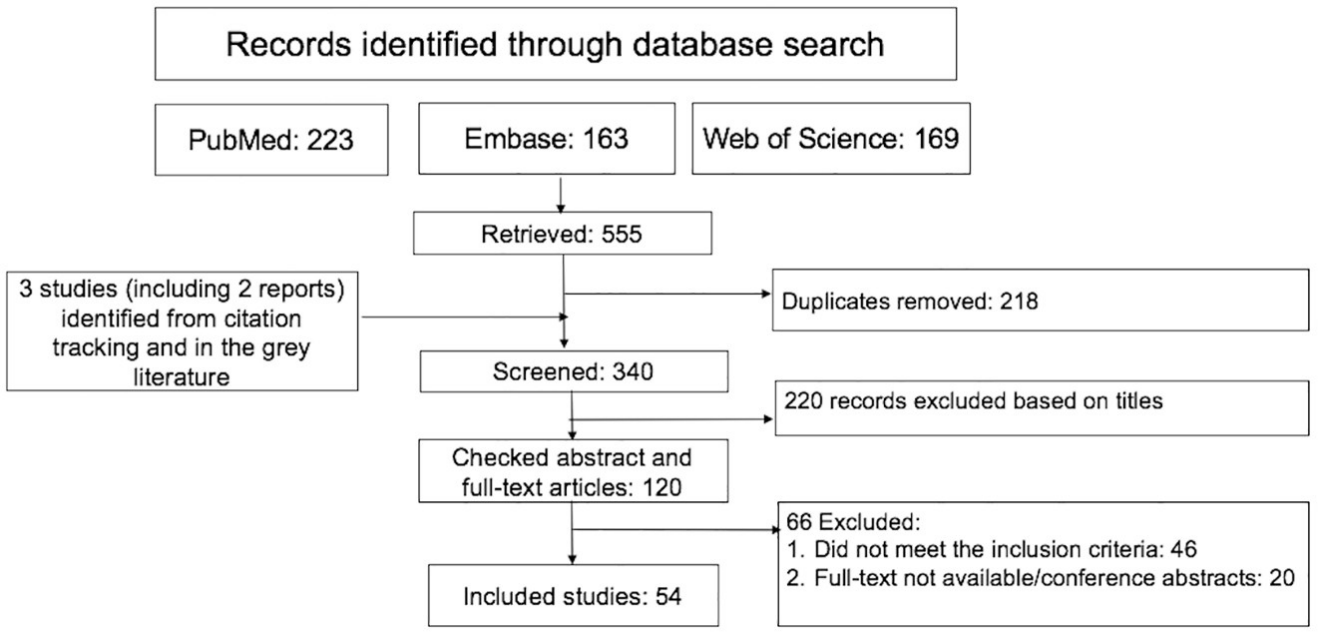
Study selection.

The flow diagram of eligible studies is shown in Fig 2. In total, 54 papers and reports (52 papers and 2 reports) covering 43 studies met the inclusion criteria. In terms of study design, 34 were quantitative, 17 qualitative, and 3 had a mixed-methods design. Of the selected quantitative/mixed methods studies, 11 reported on the prevalence of HIV and 8 on HIV incidence; 9 studies documented factors associated with HIV incidence or HIV positive status; 10 studies reported on HIV testing coverage and/or associated factors; 11 reported on alcohol and/or its association with HIV prevalence or incidence; 7 reported on antiretroviral therapy coverage/adherence/outcomes; and 1 study reported on the impact of combination HIV interventions in fishing communities. Qualitative studies addressed a range of thematic areas, and provided contextual understanding of the risk of HIV, prevention, and treatment-related factors (Table 2).

**Table 2:** Studies included in the scoping review.

Author	Publication year	Study year	Study design	Thematic areas	Sample size	Age
Abaasa et al.	2015	2012–2013	Cohort study	HIV incidence and associated risk factors	575	18–49
Abaasa et al.	2015	2009–2011	Cohort study	HIV incidence	2074	13–49
Asiki et al.	2011	2009	Cross-sectional	HIV prevalence and associated risk factors	10,188	≥ 13
Bogart et al.	2016	2014	Qualitative study	Barriers to linkage to HIV care in FC	35	≥ 18
Bogart et al.	2017	2015	Mixed-Method Study	HIV testing	1,364	≥ 18
Bonnevie et al.	2019	2016–2017	Qualitative study	Alcohol use in FC	152	15–49
Breuer et al.	2019	2016–2017	Qualitative study	Alcohol use in FC	195	15–49
Burgos-Soto et al.	2020	2016	Cross-sectional	Prevalence of HIV; HIV testing; ART coverage; viral suppression	1738	15–69
Chang et al.	2016	2011–2013	Cross-sectional	Burden of HIV, risk factors, and ART coverage in FC	17,119	15–49
Choko et al.	2018	Not specified	Pilot trial	HIV testing	135	≥ 18
IOM	2013	2013	Mixed-Methods Study	Prevalence of HIV and risk factors	1,971	15–59
Kagaayi et al.	2019	2011–2017	Serial cross-sectional [RCCS]	Trends in HIV incidence and prevalence; HIV testing; ART coverage; male circumcision; treatment outcome (viral load levels)	8,941	15–49
Kamali et al.	2016	2012–2014	Cohort Study	HIV incidence and associated risk factors	1,398	≥ 18
Kiene et al.	2019	Not specified	Cross-sectional	Association of alcohol use with HIV positive status	300	≥ 18
Kiene et al.	2019	Not specified	Cross-sectional	Association of alcohol use with risky sexual behavior	300	≥ 18
Kipp et al.	1995	1991	Cross-sectional	HIV prevalence and associated risk factors	399	≥ 15
Kiwanuka et al.	2013	2011–2012	Cross-sectional	HIV prevalence and associated risk factors	2,191	18–49
Kiwanuka et al.	2014	2011–2013	Cohort study	HIV incidence and associated risk factors	2,191	18–49
Kiwanuka et al.	2017	2011–2013	Cohort study	PAF of HIV incidence associated with alcohol	2,191	18–49
Kiwuwa-Muyingo et al.	2017	2009–2011	Cohort study	Prevalence and incidence of HIV	640	13–49
Kuteesa et al.	2020	2018	Cross-sectional	Association of alcohol use with HIV positive status	1281	15–24
Kuteesa et al.	2019	2015	Cluster randomized trial	Prevalence of HIV	862	≥ 18
Kwagonza et al.	2020	2013	Cross-sectional	Comprehensive HIV knowledge	1780	≥ 15
Kwiringira et al.	2019	Not specified	Qualitative study	HIV risk factors	12 FGD and 15 key informants	Not specified
Lubega et al.	2015	2014	Qualitative Study	HIV risk behaviors and factors	92	15–54
Lubega et al.	2015	2014	Qualitative study	HIV risk behaviors and factors	92	15–54
Lubogo et al.	2019	2012	Cross-sectional	Utilization of safe male circumcision service	369	18–54
McArthur et al.	2013	2008–2010	Qualitative study	HIV status disclosure and sexual risk behavior	20	14–48
Mafigiri et al.	2017	2013–2014	Cross-sectional	HIV prevalence and risk factors; HIV testing	792	15–24
Matovu et al.	2020	2019	Feasibility and acceptability study	HIV testing	298	15–24; ≥ 25
Mbonye et al.	2016	2014	Qualitative study	Perceptions of HIV and safe male circumcision	40	≥ 18
Mugisha et al.	2010	2007	Cross-sectional	HIV testing	127	16–44
Nakiire et al.	2020	2013	Cross-sectional Secondary data analysis from Lake Kyoga behavioral survey	HIV testing	134	15–59
Nevin et al.	2015	2013	Qualitative study	Perceptions of HIV and safe male circumcision	67	≥ 18
Ngabirano et al.	2020	Not specified	Cross-sectional	HIV risk behavior	145	13–19
Omooja et al.	2019	2016–2017	Cross-sectional	HIV treatment outcomes	1,169	≥ 15
Opio et al.	2013	2010	Cross-sectional	Prevalence of HIV and risk factors; HIV testing	911	15–59
Opio et al.	2010	2010	Mixed-method Report	Prevalence of HIV and risk factors; HIV testing	911	15–59
Pearson et al.	2013		Qualitative study	HIV risk factors	78	Not specified
Pickering et al. 1997	1997	Not specified	Cross-sectional	HIV risk behavior	80	17–52
Rosen et al.	2019	2017–2018	Qualitative study	Barriers and facilitators of ART adherence	25	Not specified
Sabri et al.	2019	2010–2011	Cross-sectional	HIV risk behaviors; Intimate partner violence	14,464	15–49
Seeley et al.	2012	2009–2011	Cohort study	HIV incidence and risk factors	1,000	13–49
Sileo et al.	2019	2015	Cross-sectional	Association of HIV fatalism and transactional sex	91	≥ 18
Sileo et al.	2016	2012	Qualitative study	Alcohol use in FC	50	≥ 18
Sileo et al.	2018	2016–2017	Cross-sectional	Intimate partner violence and HIV risk	115	≥ 18
Sileo et al.	2019	2016–2017	Cross-sectional	ART adherence and substance use	300	≥ 18
Sileo et al.	2019	2016–2017	Qualitative study	ART adherence and alcohol	30	≥ 18
Sileo et al.	2019	2016–2017	Qualitative study	Masculinity and engagement in HIV care	30	≥ 18
Sileo et al.	2019	2016–2017	Cross-sectional	Barriers and facilitators of ART adherence and clinic attendance	300	≥ 18
Tumwesigye et al.	2012	Not specified	Cross-sectional	Alcohol use and risky sexual behavior in FC	475	≥ 18
Tumwine et al.	2019	2016–2017	Qualitative study	Barriers to accessing HIV treatment care and services	57	Not specified
Tumwine et al.	2020	2016–2017	Qualitative study	Social support and access to HIV treatment, and care services	57	Not specified
Westaway et al.	2009	2004–2005	Qualitative Study	Reasons for low educational levels in fishing communities	Not specified	Not specified

ART: Antiretroviral therapy; FC: Fishing communities

### Characteristics of fishing communities in Uganda

#### Young population

Uganda has one of the youngest populations in Sub-Saharan Africa and in the world [24]. In line with the country’s demographic profile, fishing communities are characterized by a relatively young population. The proportion of respondents aged 18–24 years old in this review ranged from 19% to 44.9% [25–33]. In other studies, 33.8% of the respondents were 15–24 years old [9], 14.9% were 15–19 years old, [34] and 31.5% [35] and 41.3% were 13–24 years old [28]. Pearson et al. [36], describe how many of the fish landing sites along Lake Victoria have grown rapidly over the past 30 years, attracting many young people in search of income from fishing, fish trading or other related activities. All the landing sites are ethnically diverse, as people migrate from all over Uganda and from neighboring countries in the hope of making a living and, often, an independent lifestyle away from family ties.

#### Low educational level

The review revealed a relatively low education attainment among the fishing communities. The proportion of respondents with primary level education or less ranged from 41.9% to 95.3% [16, 18, 27–31, 33, 34, 37–45]. Westaway et al.’s 2009 qualitative enquiry in three fishing communities, Nanyolo (Lake Kyoga), Mhinga (Lake Victoria), and Kitanba (Lake Victoria), show that children in the fishing communities dropped out of school for paid work or due to social pressure (often in relation to fishing), household chores, illness of another member in the household, pregnancy, early marriage, lack of money, lack of intellectual ability, long distance from school, and the poor quality of local government schools (absenteeism, teachers lacked professionalism, poor punctuality, etc.) [46]. Similarly, Pearson et al.’s qualitative study life histories of young people who migrated to the fishing communities [36] revealed that the majority of school dropouts did not complete secondary or primary school partly due to lack of funds and pregnancy (for women). Some also had children to support from previous or current relationships and therefore, migrated to fishing landing sites for employment.

#### Occupational mobility

Mobility has been commonly cited in the literature as a risk factor for HIV transmission. A substantial proportion of residents in fishing communities, ranging from 10.6% to 51.5%, lived in the fishing communities for less than a year, while 37.6% to 59.2% lived in these communities for up to five years [25–27, 47]. Kiwanuka et al. found that people in the fishing communities frequently moved between the different landing sites and islands on Lake Victoria with up to 47% of men and 25% of women reporting being away from home for at least two days in the previous month. Mobility was mainly driven by the seasonal fish catch and could result in fishermen being away for three to six months at a time.

#### Comprehensive knowledge of HIV/AIDS

**Fig 3:**
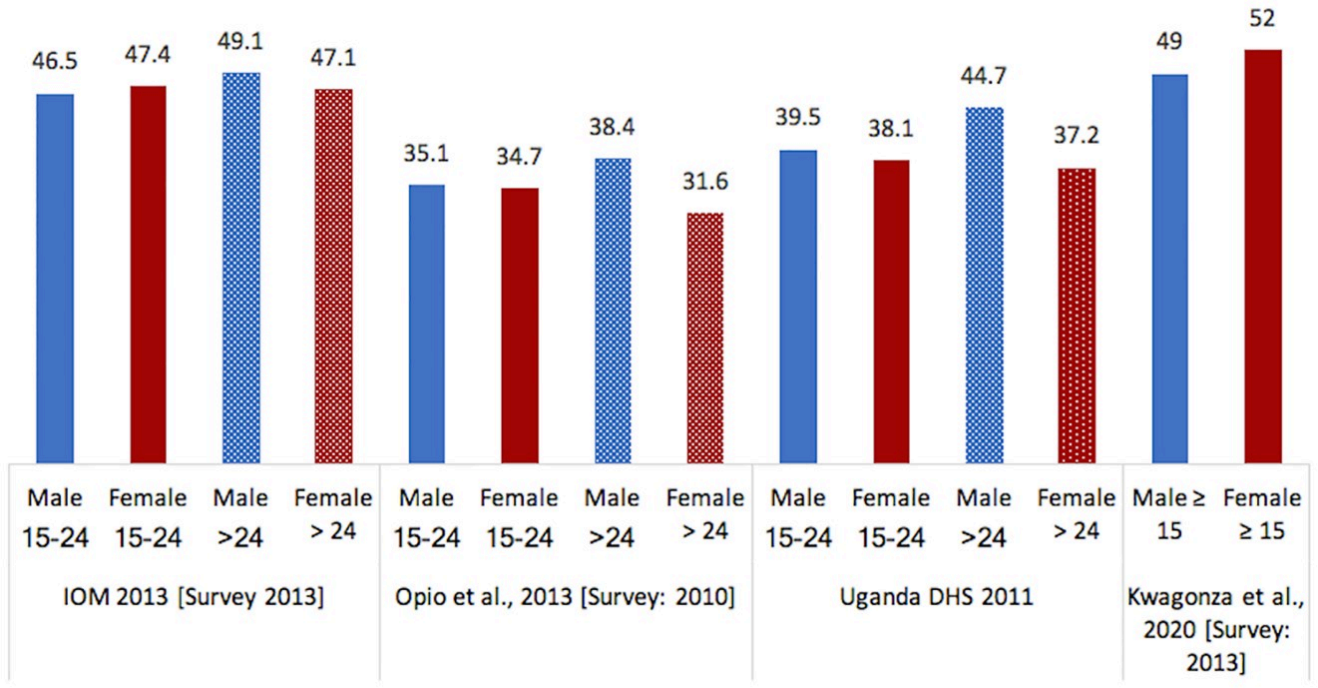
Comprehensive knowledge of HIV/AIDS. DHS: Demographic Health Survey. The numbers represent the prevalence reported as percentage (%).

The level of HIV knowledge among fisherfolk appears to be similar to that documented among the general Ugandan population (Fig 3). HIV/AIDS knowledge from Opio et al.’s [29] 2010 study of 46 fishing landing sites around the Lake Victoria basin and IOM’s 2013 study of 42 fishing communities in 6 Ugandan districts [32] were compared with results from the 2011 Uganda Demographic Health Survey (UDHS) [48]. In Opio et al.’s [29] and the IOM’s studies [32], comprehensive knowledge of HIV/AIDS was measured using five individual indicators of HIV/AIDS knowledge: 1) people can reduce the chances of getting AIDS by using a condom every time they have sex; 2) people can reduce the chances of getting AIDS by having sex with just one partner who is not infected and who has no other partners; 3) people cannot get AIDS from mosquito bites; 4) people cannot get AIDS from sharing food with a person who has AIDS; and 5) a healthy-looking person can have AIDS. Comprehensive knowledge of HIV/AIDS in the UDHS included similar items, except for item 2, which was replaced by the item “AIDS cannot be transmitted by supernatural means”. In a recent paper that assessed the comprehensive knowledge of HIV prevention among fishing communities of Lake Kyoga in 2013, Kugonza et al. (2020) found that 51% (n = 899) had comprehensive knowledge on HIV prevention. The study also found that fishermen who lived > 5 km away from a health center (46%, n = 324) were less likely to have comprehensive knowledge on HIV prevention than those who lived within a 5km radius from a health center (54%, n = 572). There was no significant difference in the level of comprehensive knowledge on HIV prevention between males and females [34].

### What is the prevalence and incidence of HIV infection in Ugandan fishing communities? (RQ 2)

#### HIV prevalence in fishing communities

**Fig 4:**
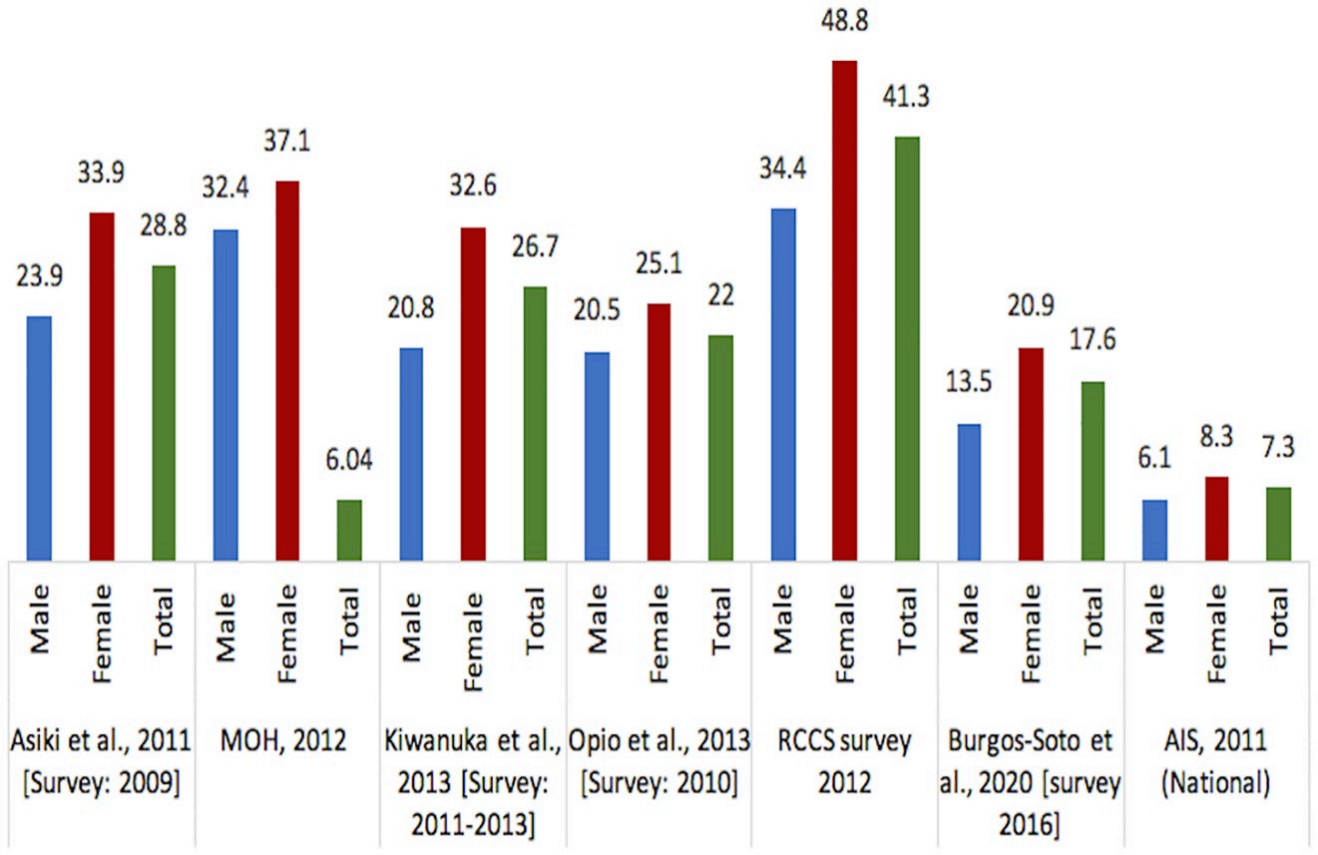
Prevalence of HIV in fishing communities. AIS: AIDS Indicator Survey (national data from the general population); UPHIA: Ugandan Population-based HIV Impact Assessment 2016–2017. The numbers represent the prevalence reported as percentage (%).

As illustrated in Fig 4, HIV prevalence in fishing communities is very high, however, it varies greatly by study ranging from 22%–37% [9, 17, 18, 27, 29, 33, 35, 47, 49] to as high as 40% in some communities [17]. These figures are 3–5 times higher than the national average prevalence for adults aged 15–49 years documented in the last two national surveys. The 2011 Ugandan AIDS Indicator Survey reported a prevalence of 7.3% [17, 50], while the 2016–2017 Ugandan Population-based HIV Impact Assessment reported a prevalence of 6.0% [51]. Concordant with data in the general population, HIV prevalence in the fishing industries is higher in women. It is important to note that this data is not limited to female fishers but also includes women working in fishing related areas such as fishmongers/factory agents, food vendors/trading, boat crew, artisanal processors, transporters, bar owners, housewives, etc.

**Fig 5:**
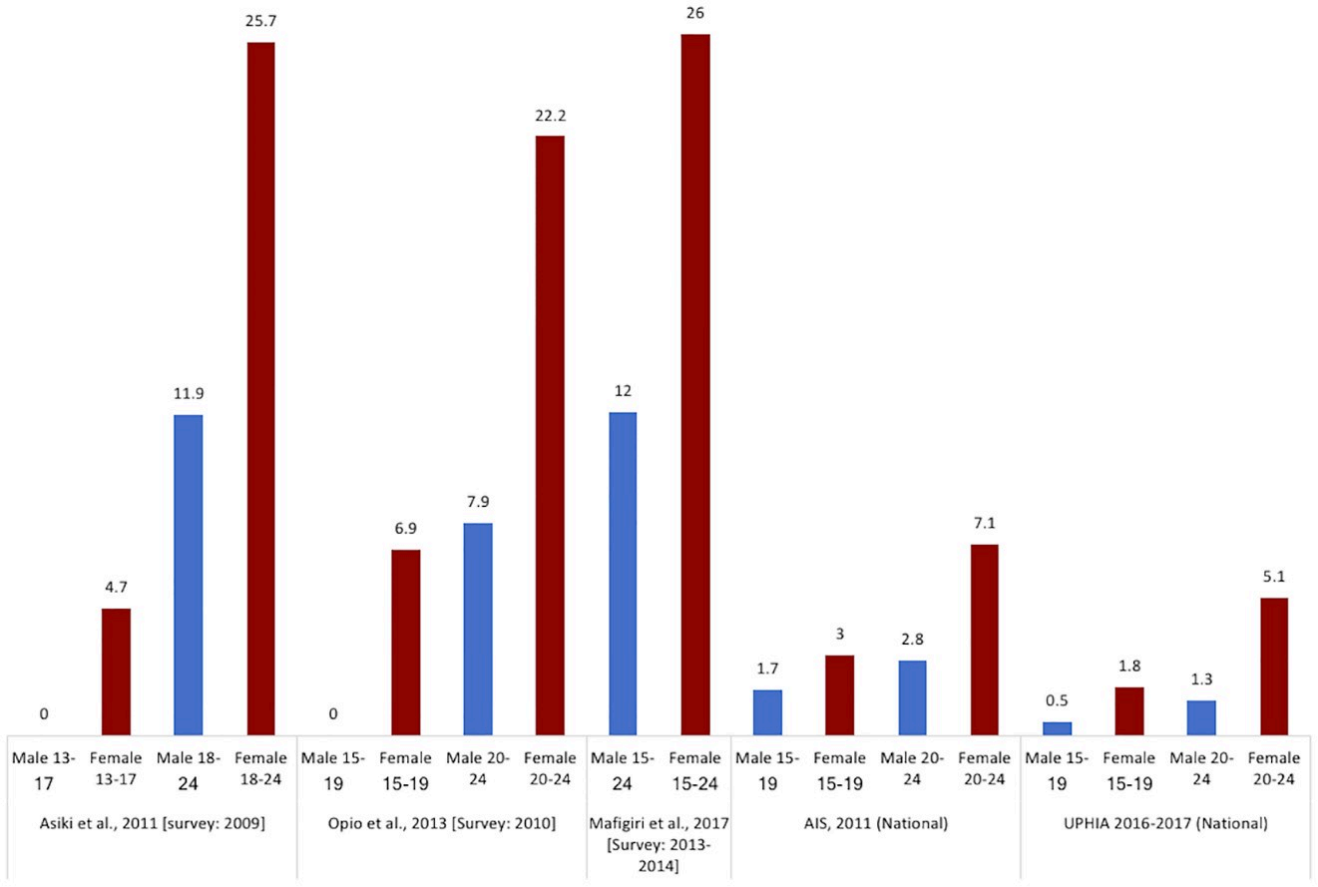
Prevalence of HIV among young people in fishing communities. AIS: AIDS Indicator Survey (national data from the general population); UPHIA: Ugandan Population-based HIV Impact Assessment 2016–2017. The numbers represent the prevalence reported as percentage (%).

*HIV prevalence among youth*. Information on HIV prevalence among young fisherfolk in the fishing communities is very limited, since only a couple of studies include those aged 15–24 years. Nevertheless, evidence from the few studies available indicate that HIV prevalence among young fisherfolk is at least two times higher than HIV prevalence among young people in the general population [51]. According to the Uganda Population-based HIV Impact Assessment (UPHIA), HIV prevalence among young people in Uganda was 2.1% (3.3% among older adolescent girls (15–19 years) and young women (20–24 years) and 0.8% among older adolescent boys (15–19 years) and young men (20–24 years). However, HIV prevalence in young fisherfolk is almost twice as high as reported in UPHIA [51]. For instance, Asiki et al. [35] found an HIV prevalence of 0.0% in men aged 13–17 (n = 33) and 11.9% among men aged 18–24 (n = 252); likewise, HIV prevalence was 4.7% in women aged 13–17 (n = 43) and 25.7% in women aged 18–24 (n = 303). Opio et al. [18, 29] found an overall HIV prevalence of 10.8% among participants aged 15–24 (n = 203). HIV prevalence was much lower in younger age groups but increased with age: from 0.0% among men aged 15–19 (n = 27) to 7.9% (n = 89) among men aged 20–24 years and from 6.9% among women aged 15–19 (n = 29) to 22.4% (n = 58) among women aged 20–24. Mafigiri et al. [38] found an overall prevalence of 19.7% (n = 155) among young people aged 15–24 years old. The prevalence was higher in females than males (26.0% versus 12%) (Fig 5).

#### HIV incidence in fishing communities

Similarly, incidence of HIV is disproportionately higher in fishing communities, as compared to the 0.39% incidence in the general population (15–49 years) [51]. HIV incidence varied by study and generally by gender, ranging from 3.1 to 5.21 per 100PY in men and from 3.37 to 9.29 per 100PY in women [25, 26, 28, 52]. A much lower incidence was observed in the four most populous fishing communities in Rakai district following a rapid scale up of HIV prevention and treatment [53]. This is described later in this review.

**Fig 6:**
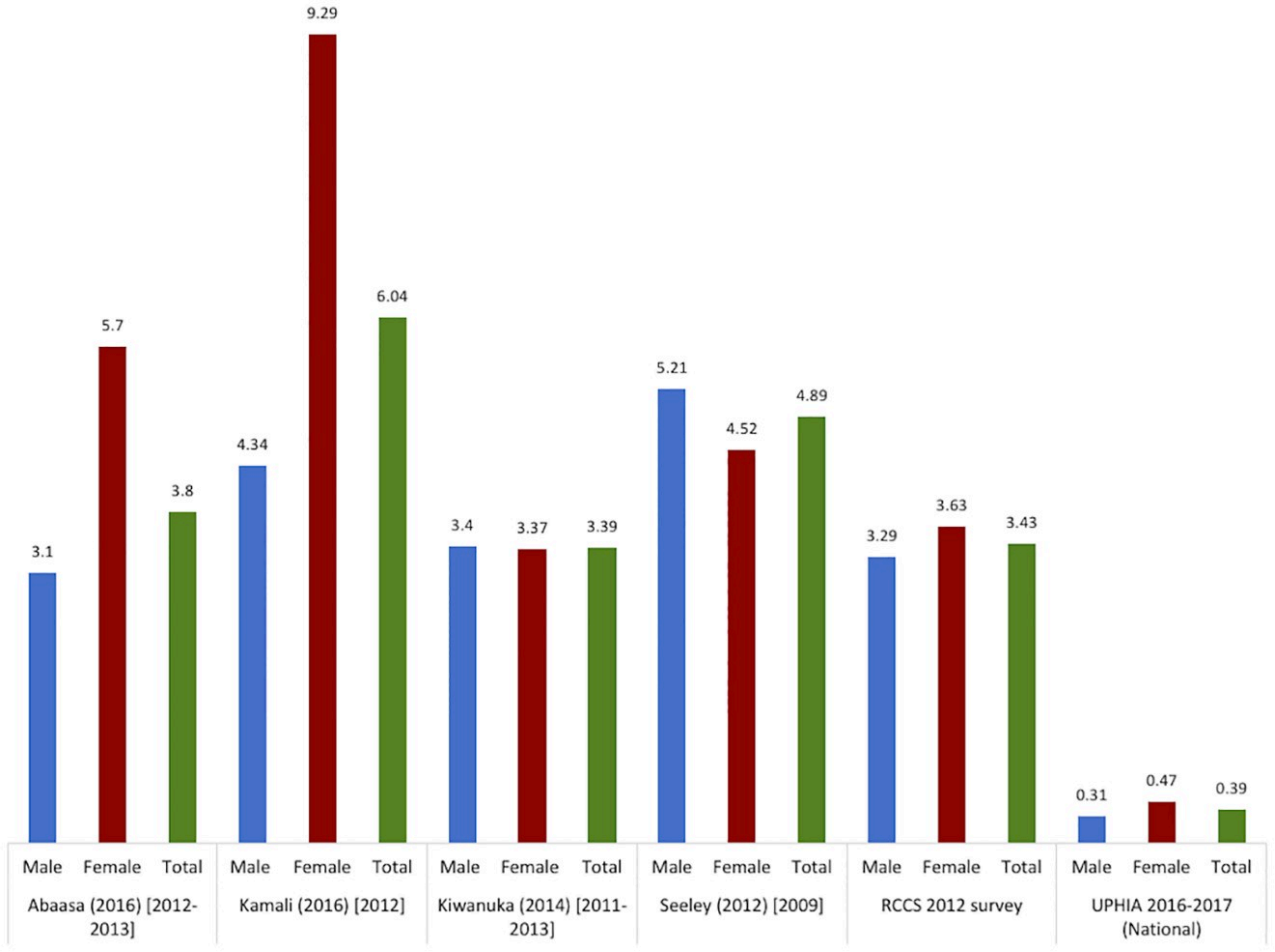
HIV incidence in fishing communities. RCCS: Rakai Community Cohort Study; UPHIA: Ugandan Population-based HIV Impact Assessment 2016–2017. The numbers represent the incidence reported as percentage (%).

In 2009, Seeley et al. [28] followed a cohort of 919 fisherfolk from five fishing communities on the shores of Lake Victoria for 18 months. The incidence rate was 4.9 (95% CI = 3.8–6.3) per 100PY. The highest rate was documented among those aged 13 to 24 years (7.5 per 100PY [5.2–11.0]), those working in bars (9.8 per 100PY [95% CI = 4.7–20.6]), the protestant religious group (8.6 per 100PY [95% CI = 5.8–12.7]), and new immigrants (6.6 per 100PY [95% CI = 4.9–8.9]) [28]. In 2011–2013, Kiwanuka et al. [52] conducted a community-based cohort study (12-month follow up) among a random sample of 2,191 participants from 8 fishing communities in three Uganda districts. The incidence rate was 3.39 per 100PY (95% CI = 2.55–4.49). The HIV incidence rate was 3.68 (2.2–6.1), 4.77 (95% CI = 2.96–7.67), and 2.45 (95% CI = 1.50–4.00) per 100PY, respectively, among those aged 18–24 years, 25–29 years, and 30 years and above [52]. Abaasa et al. found that despite similar patterns of reported risk behavior in an observational cohort study and simulated clinical trial, HIV incidence was higher in the observational cohort study than in the clinical trial (11.4 versus 3.8 per 100PY) [25]. Another study by Abaasa et al. documented an HIV incidence rate of 4.9 (95% CI = 3.8–6.3) per 100 PY at risk in a cohort study conducted from 2009 to 2011 [54]. Kamali et al. found an HIV incidence of 6.04 (95% CI = 4.36 to 8.37) per 100PY at risk based on incidence data collected between 2012 and 2014 [26]. The incidence was higher among those aged 18–24 years old 6.39 (95% CI = 3.71 to 11.0) per 100 PY at risk as compared to the 5.75 rate for those aged 25–29 years (95% CI = 2.99 to 11.05) and the 5.93 rate for those aged 30 years and above (95% CI = 3.51 to 10.05) [26]. Lastly, a prospective study from 2011 to 2017, embedded in the Rakai Community Cohort Study (RCCS), documented incidence rates of 3.29 in male and 3.63 in female respondents living in four fishing communities in Lake Victoria [53]. The RCCS is an open, population-based, observational study of HIV incidence, sexual behaviors, and health service utilization in agrarian, trading and fishing communities located in Rakai and neighboring districts of south-central Uganda [53]. Fig 6 displays the HIV incidence from the selected studies.

### What are the risk factors of HIV infection in fishing communities? (RQ 1)

#### Sexual behavior in fishing communities

**Fig 7:**
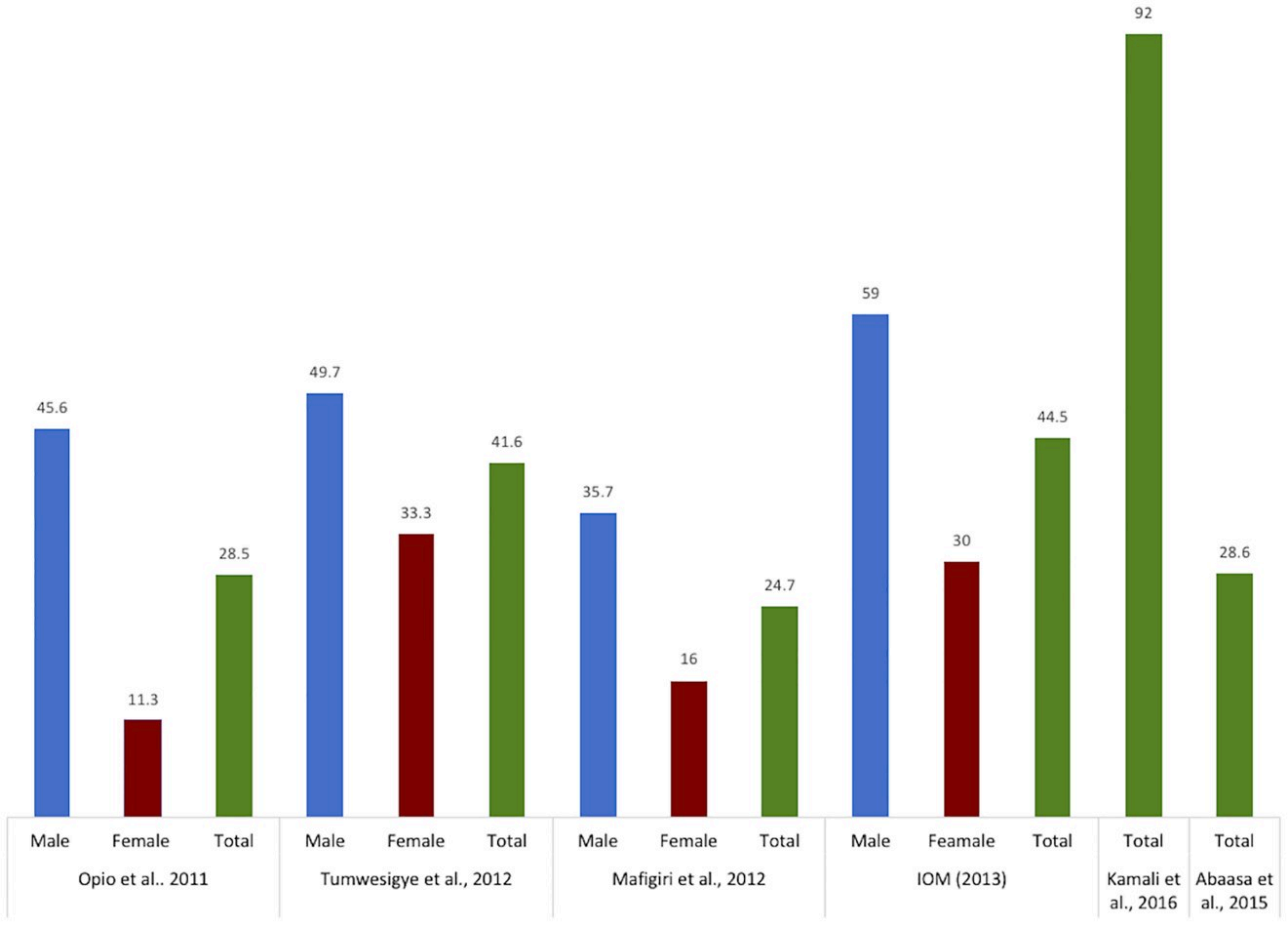
Multiple sexual partnership in selected studies. IOM: International Organization for Migration. Kamali et al. (2015): more than 2 partners in the past 12 months. Abaasa et al. (2015): more than 2 partners (no time line provided). The numbers represent the prevalence reported as percentage (%).

*Multiple sexual partnerships*. Multiple sexual partnership (MSP), defined as having two or more sexual partners, is highly prevalent in fishing communities (Fig 7). One of the earliest studies of sexual behavior conducted in a fishing community on Lake Victoria among 54 men and 26 women documented a high number of both concurrent and sequential partners. Most men reported three partners a week, with one being the regular partner, and all women regardless of their marital status reported regular sexual contact with casual and paying customers [55].

Chang et al. found that 22% of all sexually active women in the fishing communities reported multiple sexual partners in the last year compared to 12% of women in trading communities and 7% of women in agrarian communities. Similarly, 61% of all sexually active men in the fishing communities reported multiple sexual partners in the last year compared to 45% of men in trading communities and 33% of men in agrarian communities [37]. Tumwesigye et al. found that 49.7% of men and 33.3% of women in fishing communities reported MSP in the past 12 months [31]. MSP was reported by 59% of men and 30% of women in a study by the IOM [32] and by 46% of men and 11% of women in a study by Opio et al. [29]. Abaasa et al. found that 28.6% of participants reported having more than 2 partners, however, the timeline and distribution by sex was not provided [25]. In a study by Kamali et al., 92% of participants reported more than 2 partners in the past 12 months [26] while in Seeley et al. 38% reported 2 or more sexual partners in the past 3 months [28]. Asiki et al. studied sexual behavioral characteristics and found that 87% of the participants in fishing communities were sexually active in the past 3 months, reporting up to 12 different partners [35]. Forty-six percent of male and 15% female respondents reported MSP; a high number of these partners were described as “new” partners (37%) [35]. In a recent study by Matovu et al., 43.7% of young men (20–24 years) and 34% of young women reported three or more sexual partners in the past three months [56]. Ngabirano et al. found 42% and 33.4% sexually active adolescent (13–19 years) males and females reported at least three lifetime sexual partners [45].

In terms of the link with HIV, Mafigiri et al. found that the odds of being HIV positive increased with the number of sexual partners in the past 12 months. Those with one partner had an AOR = 5.0; 95% CI = 1.33–15.80 as compared to those with 2 or more partners AOR = 11.0; 95% CI = 3.04–36.72 [38]. Kamali et al. reported that having more than two sexual partners in the past 12 months was associated with increased risk of becoming infected with HIV (RR = 19.29; 95%CI = 4.95–165) and Seeley et al. found a similar trend, though the association was not statistically significant (RR = 3.0; 95% CI = 0.8–10.8) [28].

*Unprotected sex*. Evidence from prior studies suggests a high prevalence of unprotected sex among the fisherfolk. One of the lowest proportions of consistent condom use was found in a study by Burgos-Soto et al. that included 1738 adults (959 women, 779 men) in which only 5.6% of men and 4.6% of women reported always using a condom during sexual intercourse [9]. In a study by Pickering et al., women reported condom use in 93% of the 982 contacts with casual partners and never with their 689 regular contacts. Among men, condom use was reported in 67% of contacts with new and 61% of casual contacts, but never with regular partners [55]. In other studies, the proportion of condom use at last sex ranged from 21.2% to 40.6% [27, 32, 45] among women, and from 29.2% to 52.4% [27, 32, 45] among men. Consistent condom use during high-risk sex in the past 12 months was 47.2% [32] and 40.8% [29] among women and 55.8% [32] and 46.7% [29] among men, while condom use at last high-risk sex was 35.5% [32] and 47.1% [29] among women, and 40.8% [32] and 59.3% [29] among men. Consistent condom use with new partners was 49.2% and 9% in Abaasa et al. [25] and Asiki et al. [35], respectively. Mafigiri et al. found that only 6.8% of men and 1.3% of women aged 15–24 years used condoms consistently in the past 12 months. The figure was even lower (0.6%) among those who were HIV positive [38]. Asiki et al. also found low levels of consistent condom use among HIV-positive fisherfolk; 11% among men and 7% among women [35]. In a study by Kamali et al., unprotected sex during the last and/or previous three months was reported among 61% of the participants and was associated with increased risk of acquiring HIV [Risk Ratio (RR) = 3.11; 95%CI = 1.27–7.66] [26].

**Fig 8:**
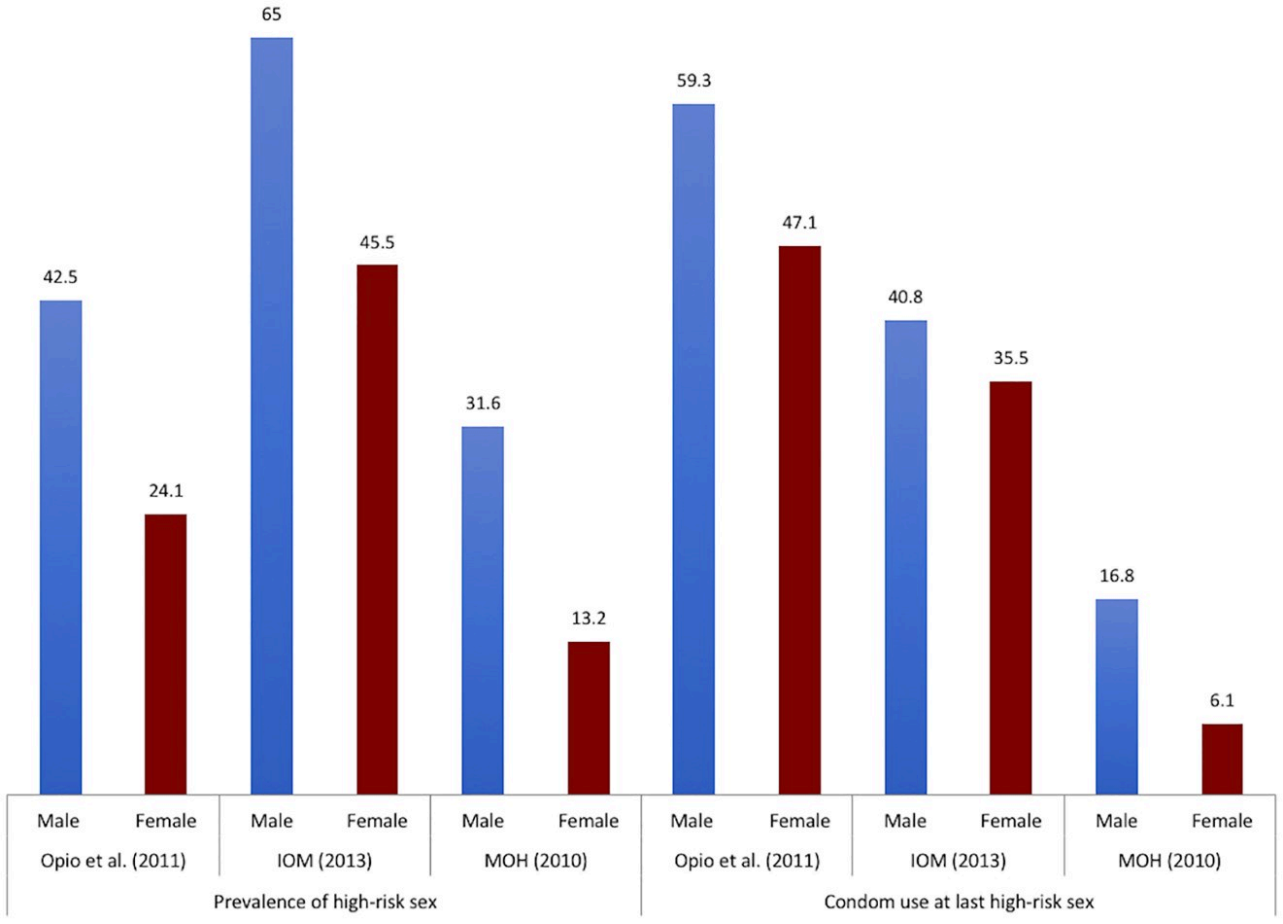
Prevalence of high risk sex and condom use at last high risk sex. IOM: International Organization for Migration. MOH: Ministry of Health. The numbers represent the prevalence reported as percentage (%).

Fig 8 displays the prevalence of high-risk sex (defined as having sexual intercourse with a non-marital partner/non-cohabiting partner) and condom use at last high risk sex in fishing communities [29, 32] and in the Ugandan general population [57]. The prevalence of high risk sex is higher in men than in women, and higher in fishing communities than in the general population. The trend remained similar for the proportion of condom use at last high-risk sex. Detailed analysis by age by Opio et al. shows that the respondents aged 20–24 years had the highest proportions of high risk sex, 56.5% of men and 32.8% of women (data for age group 15–19 are not available) [29].

*Commercial/transactional sex*. Commercial sex, as well as transactional sex (which involves the exchange of sex for gifts, fish, sugar, favor, or other items other than money), is highly prevalent in fishing communities. Pickering et al. documented that the exchange of cash was commonly reported in a high percentage of sexual contacts with casual partners (84% and 77% respectively). In contrast, for regular partnerships, a higher proportion of women reported receiving a gift (75%) and/or cash (20%) than men (5% for either gift or cash) [55].

Asiki et al. [35] found that study participants among the fishing communities had either received or given gifts such as fish (31%) or clothing (37%) in exchange for sex. Exchange of fish for sex accounted for 21% and 41% of men and women, respectively, while clothing accounted for 49% and 37% of men and women respectively.

**Fig 9:**
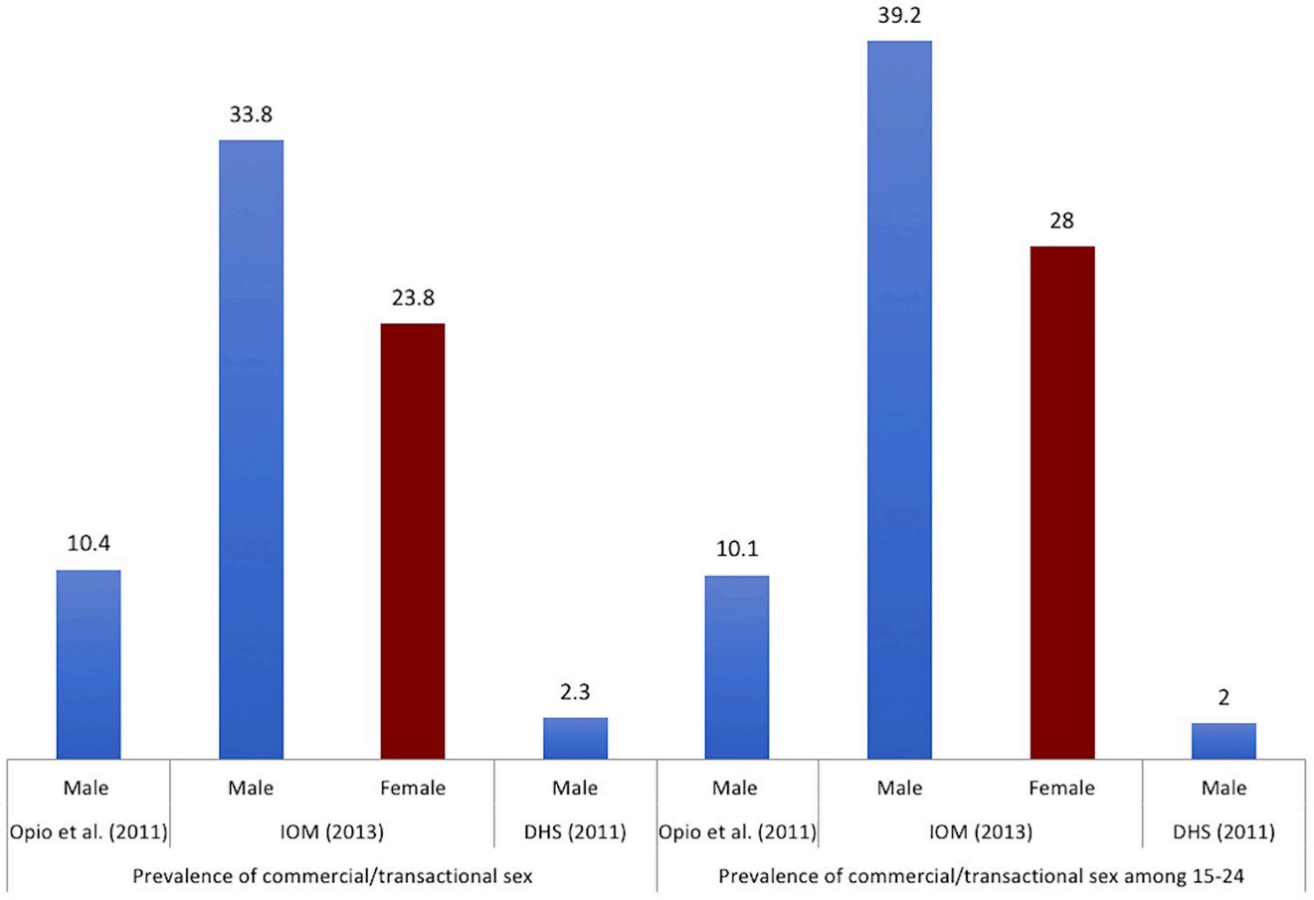
Commercial/Transactional sex in the past 12 months. IOM: International Organization for Migration. DHS: Demographic Health Survey. The numbers represent the prevalence reported as percentage (%).

Commercial and transactional sex in the previous 12 months was studied by Opio et al. [29] where they found that 10.1% of men aged 20–24 were engaged in transactional sex in the past 12 months (did not report on women). Overall, 10.4% of all men aged 15–49 years were engaged in transactional sex, of which, 79.0% reported they did not use a condom [29]. IOM’s study found that 15–24 year olds were the highest proportion of respondents who reported transactional sex in the past 12 months; 39.2% and 28.0% of men and women respectively. Overall, 33.8% of men and 23.8% of women of all ages (15–59 years) reported having had transactional sex in the past 12 months. Among men, 44.1% did not use a condom the last time they engaged in transactional sex and 56.3% reported not using condoms consistently when they engaged in transactional sex [32]. In both studies, further analysis, when cross tabulated with marital status, demonstrated that those who were widowed/separated/divorced were more likely to engage in transactional sex [29, 32] than those who were married or single. As shown in Fig 9, commercial and transactional sex is significantly higher in fishing communities as compared to the general population (data from Ugandan DHS (2011) [48]).

*Sexual risk behavior*: *Insights from qualitative studies on sexual risk behaviors*. Evidence from qualitative research corroborates findings from quantitative studies showing high prevalence of risky sexual behavior in fishing communities, and highlights a range of socio-cultural, economic, and structural factors that interplay at different levels (individual; interpersonal; community; broader policy and economic environment) to shape the sexual behavior and risk factors for HIV in Ugandan fishing communities. These include at the individual level, high consumption of alcohol, low risk perception of HIV, misperception about HIV transmission, fatalistic attitude that HIV is unavoidable, and easily disposable income. Weak family ties among fisherfolk, occupational mobility, peer pressure, non-disclosure of HIV status, continuing normal sexual practices following HIV diagnosis to conceal HIV status, and the wide availability of alcohol/bars/lodges for sexual encounters were among the factors identified at the interpersonal and community levels. Poverty and lack of enforcement of protective policies such as restrictions on alcohol use and promotion of access to education were identified as influencing factors at the broader policy and economic level [44, 58–63].

*Other sexually transmitted infections among fishing communities*. Asiki et al. [35] reported a 4.3% prevalence of active syphilis in the population with no significant difference between gender. However, multivariate analysis revealed that sexually transmitted infections (STIs) were independently and significantly associated with younger people in both genders. Furthermore, as shown in Table 3, in a study of sexually experienced men aged 20–24 by Opio et al. [18], 15.7% reported having had abnormal genital discharge in the past 12 months, 18.0% had genital sores/ulcers, and 19.1% self-reported a history of STIs. In the same study, 31.0% of women aged 20–24 also self-reported a history of STIs. However no data was available for the 15–19 age group. Overall, history of STIs was reported most frequently in people aged 20–24 years as compared to the other age groups. The IOM study documented that 23.4% of men aged 15–24 self-reported STIs [32]. Approximately 17.0% of men and 30.7% of women had abnormal genital discharge, while 15.3% of men and 32.4% of women aged 15–24 had genital sores/ulcers in the past 12 months. Roughly 23.0% of men and 41.0% of women aged 15–24 years reported having had STIs/abnormal genital discharge and sores/ulcers in the past 12 months. Kamali et al. [26] reported that 56% of participants self-reported STIs (current and/or previous three months), while Abaasa et al. [25] reported prevalence of 24.3% and 27.6% for self-reported genital discharge and self-reported genital sores, respectively. These results were, however, not broken down by age and gender. Seeley et al. found that HIV infection was independently associated with genital discharge/sores (current or previous 3 months) [adjusted Hazard Ratio (aHR) = 2.8; CI = 1.6–5.0] [28].

**Table 3:** Percentage of self-reported history of STIs, abnormal genital discharges and sore/ulcer in the past 12 months among fishing communities.

	Opio et al. (2011) [29]		IOM (2013) [32]		Abaasa et al. (2016) [25]	Kamali et al. (2016) [26]
	Men (20–24, n = 89)	Women (20–24, n = 58)	Men (15–24, n = 295)	Women (15–24, n = 293)	Total (18–48, n = 210)	Total (at least 18 years, n = 490)
STIs (self-reported)	19.1	31.0	23.4	NA	NA	56
Abnormal genital discharges	15.1	NA	17.3	30.7	24.3	NA
Genital sore/ulcer	18.0	NA	15.3	32.4	27.6	NA
STIs, genital discharge and sore/ulcer	29.2	NA	23.4	41.0	NA	NA

NA: Not applicable; STIs: Sexually transmitted infections

#### Duration of residence in fishing community

Shorter duration of residence in the fishing community was associated with higher risk of acquiring HIV [25, 26, 28]. Abaasa et al. found that participants who lived in the same fishing community for more than 5 years were less likely to acquire to HIV compared to those who lived there for less than a year (RR = 0.3; 95%CI = 01–0.8) [25], while Kamali et al. reported that those who had lived in the community for less than a year had more than a seven-fold risk of acquiring HIV compared to those who had lived there for more than 5 years (RR = 7.64; 95%CI = 2.36–24.6) [26]. Similarly, Seeley et al. found a higher risk of HIV infection in participants who resided in the community for fewer than five years compared to those who stayed more than five years (RR = 2.14; 95%CI = 1.11–4.12) [28].

#### Education level

Two studies documented an association between education level and HIV prevalence and incidence. Kiwanuka et al. found that HIV prevalence decreased with increasing levels of education: 39.8% among those with no formal education, 28.6% for primary education and 19.8% for post-primary education [27]. In their cohort study, Abaasa et al. found that respondents with no education had higher HIV incidence compared to those with primary or higher levels of education (aRR = 3.1; 95%CI = 1.3–7.7) [25].

#### Male circumcision

The prevalence of male circumcision in the fishing communities varied across studies and time period. Opio et al.’s 2010 survey among 46 fishing communities found that 40.0% of males were circumcised (n = 559). The highest rates were observed among young people; 49.4% in the 20–24 group (n = 89) and 42.9% in the 15–19 group (n = 28) [29]. Kagaayi et al. documented a circumcision prevalence of 65% among male residents of four fishing communities in Rakai district in 2016–2017, an increase from 35% in 2011–2012. Male circumcision was strongly protective, decreasing the risk of HIV acquisition by around 54.0% [53]. Burgoso et al. documented a circumcision prevalence of 56% among 779 males in a cross-sectional household-based survey in 12 fishing communities surrounding Lakes Georges and Edward [9]. Mafigiri and al. documented a rate of 53.8% among youth aged 15–24 years in Kasensero, Rakai district (n = 134); the proportion of circumcised youth was higher among HIV-negative males (n = 122; 58%) than HIV-positive males (n = 12; 27.9%) [38]. The lowest prevalence was documented in a study by Lubogo et al. [64]; 8.4% (31/369) of the participants had been circumcised in a safe male circumcision designated health facility. Factors associated with circumcision included peer support (AOR = 5.88; 95%CI = 1.66–20.00) and perceived procedural safety of circumcision (AOR = 6.8; 95% CI = 2.16–21.17).

Findings from qualitative research have identified improved hygiene, HIV prevention, and belief that male circumcision improves sexual performance as facilitators for the uptake of male circumcision in fishing communities, while barriers included increased libido in men, post-surgical abstinence, lost income during convalescence, lengthier recovery due to occupational hazards, and fear that foreskins are sold after their removal. Some local beliefs and practices surrounding circumcision increased the risk of HIV transmission. These included the beliefs that contact with vaginal fluid aid circumcision wound healing and the belief that the first sexual partner after circumcision should be a person other than one’s spouse [65, 66]. Some fisherfolk believed that male circumcision conferred total protection against HIV and STIs despite the information given by health providers to address this misconception [66].

#### Alcohol use

The high prevalence of HIV in fishing communities is influenced by the excessive use of alcohol, a deeply rooted practice in these communities. Evidence from a recent study indicates that alcohol use accounts for 64% of new HIV infections in fishing communities in Uganda. [67]. The prevalence of alcohol use in the selected quantitative studies has been consistently high; however, comparison across studies is difficult due to differing measurement methods of alcohol use. Tumwesigye et al. found that 62% of men and 52% of women in two fishing communities around Lake Victoria reported harmful use of alcohol in the past month [31]. Kiwanuka et al. reported a 52.9% prevalence of alcohol use at baseline (past three months) [27] and 53.5% at follow-up (past twelve months) [52]. Of the 53.5% who reported drinking at follow-up, 24.4% drank occasionally (two days a week or less) and 29.1% drank regularly (3–7 days a week). Kamali et al. documented that the prevalence of alcohol use in the past month was 71% [26]. In Sileo et al. 46.7% of male fisherfolk reported drinking and 30.7% were described as hazardous drinkers [41].

A survey of adolescents aged 13–19 years in four villages located near Lake Edward or Lake George found that 46.6% of adolescents reported a history of drinking alcohol [45]. A recent survey among young people (15–24 years) in fishing communities in Koome found that 659 (51.4%) reported having drunk alcohol, 248 (19.4%) had 12-month-AUDIT (Alcohol Use Disorders Identification Test) ≥ 8, and 261 (20.5%) had whole-blood PEth 16:0/18:1 concentration ≥ 20 ng/mL, indicating significant consumption [16].

Evidence suggests that consumption of alcohol is associated with increased risk of being or becoming HIV positive and increased risk of engaging in risky sexual behaviors including unprotected sex and involvement in transactional sex [27, 28, 31, 40]. Sex under the influence of alcohol was independently associated with increased risk of HIV seroconversion (RR = 2.1; 95% CI = 1.2–3.6) [28]. In Kiene et al. (2019b) [40], 18.7% of fisherfolk reported the co-occurrence of hazardous alcohol consumption patterns and alcohol-related problems, which were associated with greater odds of being HIV positive as compared to non-drinkers (AOR = 2.75;95% CI = 1.17–6.43). Tumwesigye et al. [31] found that fishermen who drank 2 or more times a week [Odds Ratio (OR) = 7.88; 95% CI = 2.05–30.24], who had an AUDIT score of 16 or more (OR = 9.04; 95% CI = 2.52–32.50), as well as those who got drunk in the previous 30 days (OR = 4.7; 95% CI = 1.74–6.24) were more likely to have engaged in transactional sex. In addition, fishermen with higher AUDIT scores were strongly associated with having had more than one sexual partner (OR = 11.9; 95% CI = 3.00–47.0). Asiki et al. found that 51%, 4% and 3% of participants reported the use of alcohol, marijuana, and khat, respectively. Among them, 35% reported they had sex under the influence of these substances. The rates of condom use were lower among daily and weekly alcohol consumers compared with non-consumers, 25% vs. 50% respectively. Alcohol consumption was associated with HIV infection in women (AOR = 1.61 95% CI = 1.20–2.17) [35]. Kiwanuka et al. reported the highest incident rates among regular alcohol drinkers (OR = 6.44; 95% CI = 4.38–9.45) [52].

Consumption of alcohol before sex and having sex while drunk was very common, with prevalence ranging from 31.4% to 52.7% [25, 32, 37, 67]. Kiene et al. (2019a) found that problematic alcohol use increased the rate of risky unprotected sex with all partners [adjusted Incident Rate Ratio (IRR) = 6.08; 95% CI = 4.30–8.60]. The increased risk was also seen among sex with casual partners and CSW/clients (IRR = 4.90; 95% CI = 3.09–7.78), and problematic alcohol use increased the odds of meeting a sex partner at an alcohol venue (OR = 2.84; 95%CI = 1.46–5.51) compared to those without problematic alcohol use [39]. In the same study, the use of commitment savings, restricted access savings account, moderated the association between problematic alcohol use and unprotected sex [39]. The use of alcohol was linked to intimate partner violence, transactional sex, and risky sexual behavior including unprotected sex and multiple sexual partnerships [30, 68].

Qualitative studies have provided socio-cultural, contextual, and structural understanding of the pervasive use of alcohol in fishing communities and its link to HIV infection through risky sexual behavior. The pervasive use of alcohol in fishing communities is a multi-layered problem. At the individual level, factors such as social influences on drinking, using alcohol to cope with stress, and lack of social responsibility were documented as reasons for risky alcohol use. At the interpersonal level, studies have documented factors such as lack of family responsibility, casual sexual relationships (including non-romantic, romantic and transactional sex, etc.), social status (purchase of alcohol evokes economic capability), and peer pressure; at the community level, the widespread availability of alcohol and the importance of the alcohol business for the local economy, culture of heavy drinking, and lack of accountability by local leaders have been documented. At the policy and societal level, lack of enforcement of national laws to restrict alcohol use emerged as an important factor [58, 60–63, 69]. The authors also documented the widespread belief that “everybody drinks” and a “life is short mentality” (due to the danger of fishing activities) as key contributors to alcohol use in fishing communities [61].

#### Fatalistic attitudes, low & desensitized risk perception of HIV

The belief that HIV acquisition and mortality is beyond one’s control, referred to as HIV fatalism, was very common among the fishermen [44, 58, 61]. Although no direct relationship between HIV fatalism and risk of HIV transmission/acquisition was documented in the reviewed studies, Sileo et al. found that HIV fatalism was significantly associated with increased likelihood of transactional sex (AOR = 3.07, 95% CI = 1.02–9.23), suggesting the possibility that HIV fatalism could contribute to HIV infection among this population [44].

Moreover, fishermen downplayed the risks and consequences associated with HIV when compared to the daily risks associated with their work such as storms, accidents onboard, and drowning. Survival in their profession took priority over prevention of HIV [58, 61]. This fatalistic attitude and low HIV risk perception was compounded by a range of factors such as widespread availability of bars and lodges, exposure to alcohol and commercial/transactional sex in fishing communities, easily disposable income of fisherfolk, and sex starvation after spending days on the lake catching fish [58, 61].

### What is the prevalence of and factors that influence HIV testing in Ugandan fishing communities? (RQ3)

Recent evidence from the Rakai Community Cohort Study (RCCS) in Rakai district, southwestern Uganda, demonstrated the viability of achieving high levels of HIV testing in fishing communities. Within the Rakai community, HIV testing coverage has increased from 68% in 2011–2012 to 98% in 2016–2017 [53]. Overall, HIV testing coverage has varied across studies and fishing communities. In Opio et al. (2010) [29], 77% of women and 62% of men reported having been tested for HIV, while in the IOM study, HIV testing coverage ranged between 82% and 90% among men and women, respectively [32]. Opio et al. [29] found younger age groups had lower HIV testing rates compared to older groups. For example, 35.7% of male participants aged 15–19 years reported having been tested for HIV compared to 61.5% among those aged 50–54. Similarly, 56.7% of female participants aged 15–19 years reported having been tested for HIV compared to 81.5% among those aged 45–49. This is in contrast to the high prevalence of HIV testing in both HIV-positive (89.5%; n = 136) and HIV-negative youth (92%; n = 435) aged 15–24 years old as documented by Mafigiri et al. [38]. The proportion of individuals that have been tested and have received their HIV test results also varied across studies; 96% in a study by the IOM [32], 92% in Matovu et al. [56], and 54.4% in a study by Opio et al. [18]. A recent study that compared home-based HIV testing and outreach event-based testing among 1,364 fisherfolk residents in Lake Victoria found that although home-based HIV testing uncovered a higher number of new HIV cases, it was associated with lower linkage to care compared to event-based testing [70]. Burgos-Soto et al. found that 93.4% of adults reported having an HIV test at least once in their lifetime and among HIV-negative adults, 81.0% reported having an HIV test within the last 12 months [9]. Another study found that peer-based distribution of HIV self-test kits was a promising HIV testing model associated with high uptake among fishermen in Bulisa, Uganda [71]. The use of a peer-led HIV self-testing model to improve HIV testing and linkage to HIV care has also been shown to improve HIV testing uptake and linkage to HIV care among 298 young people (15–24 years) and adult men (25+ years) in the Kasensero fishing community in Rakai [56]. Of the 21 individuals that tested HIV-positive, 57% (n = 12) were first-time testers. After confirmatory HIV testing was done, 10 out of 12 self-tested HIV-positive individuals were confirmed as HIV-positive, with 9 out of 10 (90%) confirmed HIV-positive individuals linked to HIV care within the first week of their HIV diagnosis [56]. In a secondary analysis of the Lake Kyoga fishing community HIV behavioral survey, disclosure of individual HIV results to a partner [AOR = 16; 95% CI = 3.6–67], residence for > 1 < 5 years [AOR = 0.04; 95% CI = 0.005–0.33], and no mobility [AOR = 3.6; 95% CI = 1.1–12] were significantly associated with utilization of couple HIV counseling and testing service [72]. (AOR = 4.91; 95% CI = 1.68–14.37).

Qualitative studies have identified a range of barriers to HIV testing including structural barriers (testing center being located very far away), provider-related barriers (lack of privacy and HIV test kits, lack of HIV testing services on the islands, inadequate number of health workers, poor referral systems, and long queues/long waiting time at the testing center), and client-related barriers (mobility of fisherfolk, irregular work schedule of fisherfolk not compatible with testing opening hours, stigma, and limited knowledge of the benefits of testing for HIV, among others) [29, 32, 43].

### Antiretroviral treatment coverage and adherence to ART (RQ 4&5)

There are remarkably few studies that have reported on ART coverage and adherence to ART among HIV-positive fisherfolk in Uganda. Overall, current evidence indicates that fishing communities have been underserved by HIV treatment and prevention services. However, a report of ART coverage in the four fishing communities included in the Kagaayi et al. study [42] indicates that ART coverage has increased from 16% (254/1598) in 2011–2012 to as high as 82% (1420/1740) in 2016–2017 following expansion of the combination HIV intervention services [53]. The ART coverage was lower in younger age groups; 28% in males aged 15–24 years compared to 87% in those aged 40–49 years and 70% in females aged 15–24 years compared to 95% in those aged 40–49 years [53]. Mafigiri et al. examined data from the 2013–2014 RCCS survey in the Kasensero fishing community and found that only 22.4% (n = 34) of the HIV-positive young people (aged 15–24 years) were receiving ART [38]. Chang et al. examined ART coverage from the 2011–2013 RCSS survey and found lower ART use (18%) among HIV-positive female residents in the fishing communities compared to female residents in the trading (33%) and agrarian communities (38%). The respective figures for HIV-positive male residents were 13%, 27%, and 24% in the fishing, trading and agrarian communities, respectively. Community ART coverage was lowest in fishing communities (median = 15%, IQR = 12–17%), compared to 40% in trading communities (IQR = 23–43%) and 31% in agrarian communities (IQR = 26–38%) [37]. In Burgos-Soto et al. 98.7% (95% CI: 96.1–99.6%) of those aware of their HIV status were on ART [9].

Quantitative data on adherence to ART and retention in care in fishing communities has, so far, been produced by a single research study conducted between October and March 2017 among 300 male fisherfolk on ART in 3 landing sites and 4 islands in Wakiso District, Uganda [41, 73]. Overall, 31% of the participants had a sub-optimal level of adherence (< 95% of ARVs taken as prescribed) and 39% missed clinic appointments in the prior year. Factors associated with ART non-adherence included younger age, lower income, and stigma (anticipated and enacted stigma). Furthermore, younger age, anticipated HIV stigma, “not” living with a partner, longer travel time to the clinic, accessing HIV care at a landing site (versus on an island) were associated with increased odds of missing clinic visits [73]. Significant interaction was found between hazardous alcohol consumption and number of pills prescribed; for example, men taking twice daily ART who also report hazardous alcohol use were nearly five times as likely to report missed antiretroviral (ARV) medication than men taking twice daily dosing who had no hazardous alcohol use (AOR = 4.91; 95% CI = 1.68–14.37) [41]. There was, however, no direct association between alcohol use (including hazardous drinking) and adherence to ART [41].

Qualitative studies have explored the barriers and facilitators to accessing HIV treatment and care services, as well as barriers and facilitators of ART adherence among the fisherfolk [69, 74–77]. Across studies, occupational mobility emerged as one of the most prominent barriers to linkage to HIV treatment and care among the fishermen. Frequent and prolonged voyage on the rivers, and the unpredictability of the duration of the trips (which largely varied according to the fish catch) increased the likelihood of fishermen running out of ARV pills [74, 76, 77]. Rosen et al. documented that there was a culture of ART sharing within occupational networks, as a coping mechanism in response to short-term treatment interruption fueled by frequent occupational mobility [74]. Sileo et al. (2019) identified alcohol consumption as one of the main barriers to ART adherence. Alcohol affected adherence through multiple pathways, including cognitive impairment (forgetting to take medication), staying out too late drinking while their ARV medication was at home, and intentionally skipping doses when drinking [69]. Barriers to accessing HIV treatment and care services included poor transport system [76], competing needs for work during clinic hours, stigma, low quality of care, and low social support [77]. With regards to facilitators, Tumwine et al. found that instrumental support from friends or family enabled access to relevant HIV services [78], while Sileo et al. found that peer support and the restorative effect of ART on the masculine roles as a worker and provider, husband and sexual partner promoted ART initiation and continued engagement in care [75].

### Treatment outcomes: Viral suppression (RQ 6)

Very few studies have examined ART outcomes among the fisherfolk. We identified 3 studies that reported on the proportion of fisherfolk on ART who have achieved viral suppression (defined as less than 1,000 copies per mL) [42, 53]. The proportions were encouragingly high; 80% (1,383 of 1,734) in Kaagayi et al. in a survey (2016–2017) conducted among HIV-positive fisherfolk in the four most populous fishing communities in Rakai District [53], 73% in a cross-sectional household-based survey in 12 fishing communities surrounding Lakes Georges and Edward in 2016 [9], and 91.7% in a cross-sectional study (2016–2017) of 1,169 HIV positive fisherfolk enrolled in a multi-site cohort of 6,000 participants of the HIV Molecular Epidemiological Study [42]. However, in the latter study, there was widespread acquired drug resistance (73.2%; 71/97) among detected cases of virological failure [42].

### Intervention studies (RQ 7 & 8)

**Fig 10:**
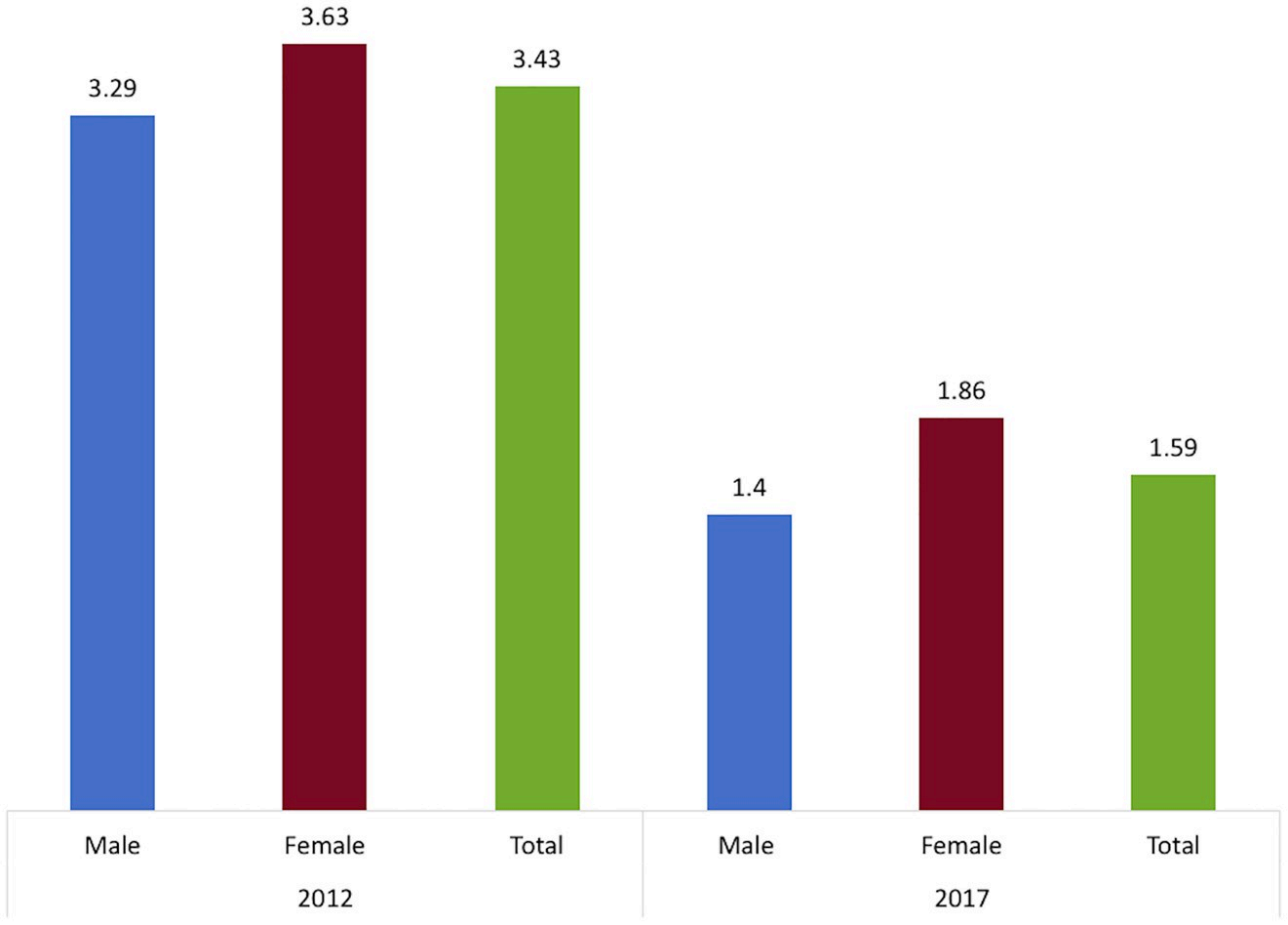
Change in HIV incidence in the RCSS. RCSS: Rakai Community Cohort Study. The numbers represent the incidence reported as percentage (%).

This scoping review revealed a gap in evidence in terms of what works for HIV prevention and treatment outcomes among the fisherfolk in Uganda. There is a dearth of published research with rigorous study designs evaluating the effectiveness of interventions related to HIV prevention or treatment outcomes. However, a recent study by Kagaayi et al. [53] reported on the impact of rapid expansion of combination HIV prevention interventions (including ART coverage and voluntary medical male circumcision (VMCC)) in the four RCCS fishing communities. This was an open population-based cohort of individuals aged 15–49 years in 40 communities located in Rakai and neighboring districts of south-central Uganda, established in 1994. In 2011, four of the most populous Lake Victoria fishing communities were added to the RCCS. Between the baseline (2011–2012) and the latest follow-up survey (2016–2017), ART coverage increased from 16% (254 of 1,598) to 82% (1,420 of 1,740), male circumcision coverage from 35% (698 of 2,011) to 65% (1,630 of 2,525), the population viral load suppression from 34% (546 of 1,596) to 80% (1,383 of 1,734), and the proportion of respondents reporting a history of HIV testing increased from 68% (2,613 of 3,870) to 96% (4,526 of 4,738). The HIV prevalence and incidence decreased, respectively from 41% to 37% and from 3.43 to 1.59 per 100PY between 2011–2012 and 2016–2017 (See Fig 10). Consistent condom use with non-marital sex partners, however, decreased from 41% (340 of 832) at baseline to 35% (387 of 1,120) at the last follow-up visit [53].

## Discussion

We conducted a scoping review to map the existing evidence on HIV infection among the fisherfolk in Uganda. The reviewed studies investigated the burden of HIV infection, HIV-related risk factors, HIV testing, ART coverage and adherence to ART, and interventions for HIV prevention to improve HIV treatment outcomes in Ugandan fishing communities.

Results from this review suggest that a complex interplay of factors conspire to enhance the risk and vulnerability to HIV infection in fishing communities, explaining the documented high incidence and prevalence of HIV in these communities. These factors align with the social-ecological model (SEM) framework, which posits that, factors at multiple levels, intrapersonal, interpersonal, community, and policy environment, interact to influence health behavior [79]. Quantitative studies that have reported on HIV incidence and prevalence in this review have largely examined individual-level factors associated with HIV prevalence and incidence in fishing communities [9, 18, 25–29, 33, 35, 37, 38, 47, 49, 52, 54]. Qualitative research has enriched quantitative studies by identifying elements relevant for understanding the dynamics of HIV transmission within the socio-cultural and structural contexts of fishing communities and across the individual, interpersonal, community, and policy environment spectrum [30, 31, 40, 44, 46, 58–63, 68, 74–77].

**Fig 11:**
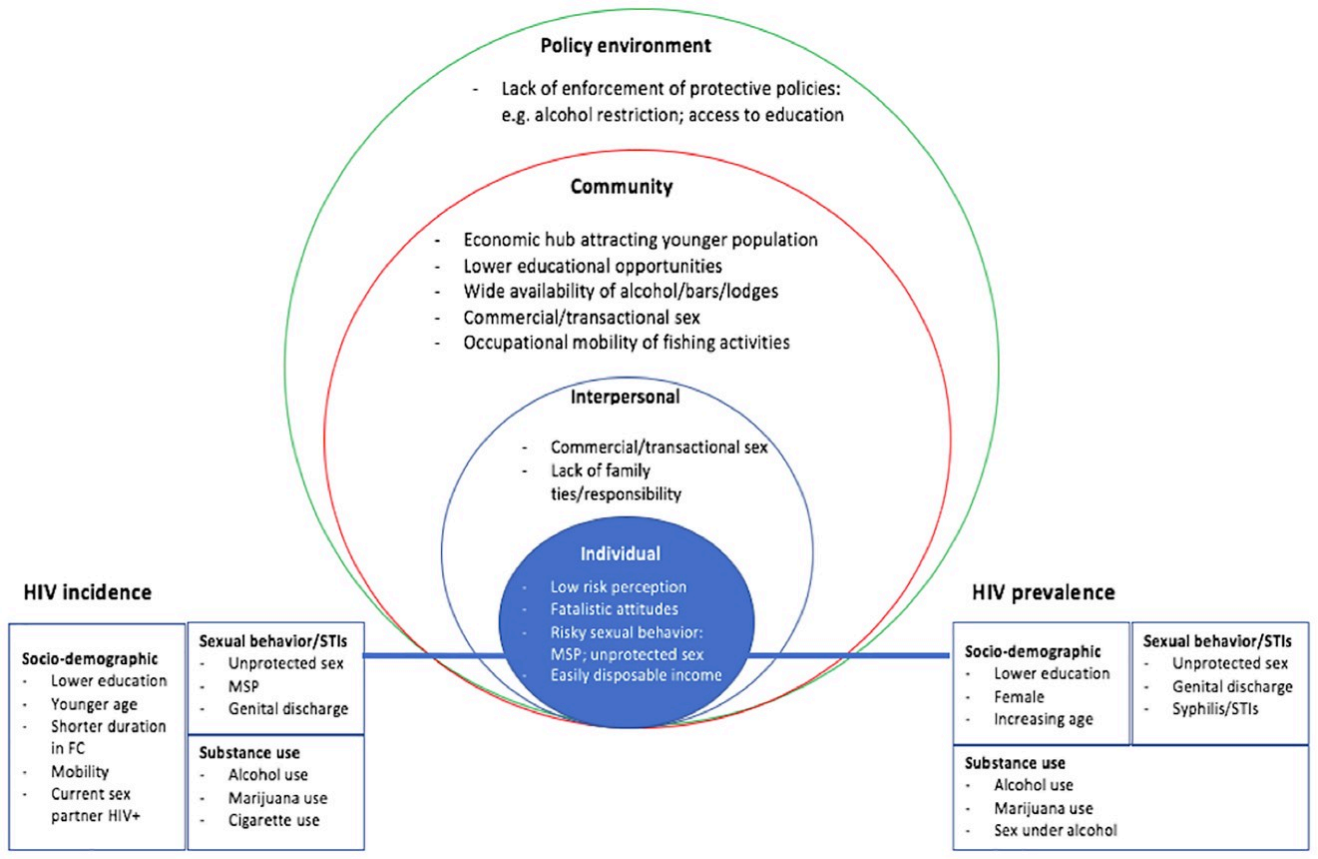
Risk factors for HIV infection in Ugandan fishing communities based on the socio-ecological model.

As illustrated in Fig 11, the individual-level factors found to be independently associated (p<0.05) with the HIV seroconversion in the multivariable analysis of cohort studies of fishing communities consisted of socio-demographic factors (lower educational levels, younger age, shorter stay in fishing communities, mobility, current sex partner HIV positive status), risky sexual behavior and STIs (unprotected sex, MSP, genital discharge), and substance abuse (alcohol, marijuana, tobacco smoking). Most of these factors were also associated with HIV positive status, with the exception that increased age was associated with higher prevalence of HIV status. From the qualitative research, low perception of HIV risk, fatalistic attitude that HIV is unavoidable, the ‘life is short mentality” resulting from the dangerous nature of fishing loaded negatively on the perception of HIV risk. Occupational mobility, substance abuse (alcohol principally), easily disposable income, and commercial/transactional sex activities were additional principal drivers of MSP and unprotected sex among residents of fishing communities [44, 58–63]. Interpersonal level factors included a lack of family ties/responsibilities of fisherfolk and peer pressure. At the community level, wide availability of alcohol/bars/lodges for sexual encounters and a predominantly young population increased HIV risk. Finally, poor enforcement of protective policies such as restricting alcohol use and promoting access to education were societal level factors [44, 58–62]. The fishing communities did not have inadequate knowledge of HIV/AIDS in contrast to the general population [17, 29, 34, 48, 50], implying that the above socio-cultural, behavioral, demographic, and structural characteristics might play a larger role in explaining the pattern of risky sexual behavior and the high incidence of HIV in fishing communities. Therefore, it is critical for prevention interventions in fishing communities to adopt a comprehensive approach that address these factors, extending from individual to the policy level factors.

Research in fishing communities is characterized by a notable lack of intervention studies, limiting our understanding of what types of HIV prevention work and improve treatment outcomes. However, recent results based on the RCCS have demonstrated the feasibility of achieving rapid expansion of combination HIV prevention interventions (including ART coverage, HIV testing and VMCC) and have shown that such an expansion is associated with reductions in both HIV incidence and prevalence in four HIV hyper-endemic fishing communities in Rakai district [53]. The incidence has, however, remained 15 times higher than the level required for achieving HIV epidemic control (e.g. 0.1 per 100PY [80, 81]). As suggested by the authors [53], epidemic control in hyperendemic communities such as fishing communities might require higher ART coverage levels and rates of male circumcision than those currently achieved in these communities. The introduction of newer/novel interventions, (such as oral pre-exposure prophylaxis (PrEP) or long-acting injectable PrEP) are other underutilized strategies [53]. It is important to emphasize that in order to optimize the impact of combination HIV prevention interventions, strategies should include both biomedical strategies (ART coverage and VMMC) and behavioral interventions that address the underlying socio-cultural, behavioral and structural factors that enhance risk of and vulnerability to HIV infection at the individual, interpersonal, community and policy levels.

In addition to the lack of intervention studies, this scoping review has revealed a lack of quantitative studies documenting adherence to ART and retention in care and their associated factors in fishing communities. A sustained high level of adherence to ART is critical to ensure not only the therapeutic and preventive benefits of ART but also to prevent emergence of drug-resistant strains of HIV [82]. To date, there was only one study that has addressed this research gap among fisherfolk in Wakiso district in Uganda, whereby a substantial proportion of participants had sub-optimal levels of adherence (31%) and missed clinical visits (39%) [41, 73]. This corroborates findings from qualitative studies indicating that fisherfolk are faced with daunting challenges (e.g. mobility, poor transportation system, irregular working schedule of fisherfolk) in accessing treatment and maintaining high levels of ART adherence [76, 77].

Programs and interventions aimed at preventing HIV and improving ART outcomes in fishing communities should consider key vulnerability factors including occupational mobility of fisherfolk with a focus on the younger members of the fishing communities. High degree of mobility and the time fishermen spend away from home are not only contributing factors to HIV infection [25–27], but also barriers to accessing prevention services such as HIV testing [43] and to accessing and adhering to ART [74, 76, 77]. Systems-level changes that promote service delivery models that accommodate lifestyles of fisherfolk are needed to address this structural barrier. Examples include integrated prevention (HIV testing) and treatment service delivery models that are directly brought to isolated fishing communities [77], or service delivery models that allow fisherfolk to send one person to collect ARV pills for the group [76]. Furthermore, young people account for a significant proportion of the population in Ugandan fishing communities. Across this review, young people have emerged as a particularly vulnerable population among the fisherfolk. Younger people tended to have higher HIV incidence, lower prevalence of HIV testing, and were more likely to miss clinic visits and to have sub-optimal level of ART adherence [28, 29, 52, 73]. HIV prevention and treatment programs need to cater to the needs of this specific population group.

This study is the first to provide a comprehensive review of the situation of HIV infection in Ugandan fishing communities. There are a few limitations worth mentioning. We excluded conference abstracts that did not have full text, resulting in a potential loss of relevant information. Another potential loss of relevant data results from the fact that articles were screened based on titles and abstracts with explicit mention of fishing communities as the target population.

## Conclusion

Fisherfolk remain the hardest hit group by the HIV epidemic in Uganda. The high incidence of HIV in fishing communities is the result of the complex interplay of factors operating at multiple levels of influence to enhance vulnerability to HIV infection. These include among many others, high rate of sexual partner change, low levels of condom use, younger age, low perception of HIV risk as compared to occupational risk of fishing, widespread culture of alcohol consumption and commercial/transactional sex and occupational mobility; all operating in the background of poverty. The recently observed downward trend of HIV incidence in selected hyperendemic fishing communities following rapid expansion of combination HIV prevention interventions (HIV testing; VMMC; ART coverage) opens a window of hope for the control of the HIV epidemic in fishing communities. However, the fact that incidence rates remained high and above the level required for HIV epidemic control despite wide coverage of ART and VMMC, is an indication that these biomedical interventions are enough. Containment of the HIV epidemic may be achievable if biomedical methods are carefully combined with behavioral change interventions addressing the underlying socio-cultural and behavioral factors that enhance risk and vulnerability to HIV infection in fishing communities. Research is urgently needed to address the existing knowledge gaps and inform policy and practice in terms of what intervention works for reducing risky sexual behavior and substance abuse, and improving uptake of HIV testing services, retention in care and adherence to ART in fishing communities in Uganda.

## Supporting information

S1 Appendix Search strategy for Embase and Web of Science databases. (DOCX)

S1 Table PRISMA checklist scoping review. (DOC)
