# Supplementary material for: HIV epidemic in fishing communities in Uganda: A scoping review
Source: PLoS One. 2021 Apr 1;16(4):e0249465. doi: 10.1371/journal.pone.0249465 (PMC8016276; doi:10.1371/journal.pone.0249465)
Supplement: S1 Appendix — (DOCX) [file pone.0249465.s001.docx]

**Searching EMBASE**

*Searches were conducted in all fields with the following search strategy*:

‘fishing communit*’/exp OR (‘fisherfolk’/de OR ‘fisherm*’/de OR ‘seafarer’/de OR ‘seamen’/de OR ‘boatman’/de OR ‘fisheries’/de OR ‘fish farm’/de) AND (‘Human immunodeficiency virus’/exp OR (‘Human immunodeficiency virus infection’/de OR ‘sexually transmitted disease’/de OR ‘sexual behavior’/de OR ‘antiretroviral therapy’/de OR ‘circumcision’/de OR ‘alcohol’/de)) AND ‘Uganda’/exp

**Searching Web of Science**

*Searches were carried out using the TS (title, abstract and keyword) field*:

(fishing community OR fisherfolk communit* OR fisherm* OR fisherfolk OR seafarer OR seamen OR boatman OR fisheries OR fish farm) and (HIV OR hiv infections OR hiv infections/epidemiology OR acquired immunodeficiency syndrome OR hiv epidemiology OR sexually transmitted diseases OR prevalence OR hiv prevalence OR incidence OR hiv incidence OR risk factors OR sex factors OR sexual behavior OR sexual partners OR health risk behaviors OR risk-taking OR risky sexual behavior OR risky behavior OR associated factor* OR predictor* OR determinant* OR unsafe sex OR circumcision, male OR male circumcision* OR health knowledge, attitudes, practice OR hiv infections/diagnosis OR mass screening OR hiv testing OR voluntary counsel* and testing OR hiv testing and counsel* OR hiv infections/diagnosis OR serologic test OR point-of-care testing* OR antiretroviral therapy, highly active OR anti-hiv agents OR patient compliance OR medication adherence OR antiretroviral therapy OR hiv control OR hiv prevention OR cross-sectional studies OR health surveys OR cohort studies OR case-control studies OR clinical study OR qualitative research OR hiv intervention) and Uganda.
